# Xanthohumol: Mechanistic Actions and Emerging Evidence as a Multi-Target Natural Nutraceutical

**DOI:** 10.3390/nu18030520

**Published:** 2026-02-03

**Authors:** Mackenzie Azuero, Camilla F. Wenceslau, Wenbin Tan

**Affiliations:** 1 Department of Cell Biology and Anatomy, School of Medicine, University of South Carolina, Columbia, SC 29209, USA; 2 Department of Biomedical Engineering, College of Engineering and Computing, University of South Carolina, Columbia, SC 29208, USA; 3 The Cardiovascular Translational Research Center, School of Medicine, University of South Carolina, Columbia, SC 29209, USA

**Keywords:** xanthohumol, NF-κB, anti-inflammation, antioxidant, Nrf2

## Abstract

Background: Xanthohumol (XN), a prenylated chalcone flavonoid derived from hops (*Humulus lupulus*), is increasingly recognized as a highly pleiotropic natural compound. Objective: We aimed to structure XN’s mechanistic hierarchy with emerging translational relevance across disease areas. Methods: We performed a comprehensive and integrative literature review of XN for its biological and translational effects across cancer, metabolic, neurological, cardiovascular, hepatic, renal, and dermatological disorders. Results: Mechanistically, XN exerts diverse bioactivities by inhibiting major pro-oncogenic and pro-inflammatory pathways, such as NF-κB, PI3K/Akt/mTOR, STAT3, HIF-1α, and selective MAPK cascades, while activating cytoprotective signaling, such as the Nrf2/ARE and AMPK pathways. Through these coordinated actions, XN modulates redox homeostasis, mitochondrial integrity, apoptosis, autophagy, ferroptosis, and inflammatory responses. In oncology, XN demonstrates broad-spectrum anticancer activity in preclinical models by inhibiting proliferation; inducing cell cycle arrest and apoptosis; suppressing epithelial–mesenchymal transition, angiogenesis, and metastasis; and restoring chemosensitivity in resistant cancers, including breast, lung, gastric, liver, and head-and-neck carcinomas. Beyond cancer, XN exhibits multi-organ protective bioactivities through antioxidative, antimicrobial, antiviral, and anti-inflammatory activities; inhibition of ferroptosis and excitotoxicity; and preservation of mitochondrial integrity. It shows beneficial effects in preclinical models of Parkinson’s disease, Alzheimer’s disease, hepatic steatosis and fibrosis, renal ischemia–reperfusion injury, cardiovascular dysfunction, skin photoaging, and atopic dermatitis. Human subject studies demonstrate that XN is safe and well tolerated, with observed reductions in oxidative DNA damage and inflammatory cytokine release. Recent advances in micellar formulations have improved XN’s systemic bioavailability and thus its translational feasibility. Conclusions: In summary, XN is a safe, multifunctional natural compound with strong potential for modulating disease-relevant biological pathways associated with cancer, neurodegenerative diseases, metabolic disorders, and inflammatory skin conditions. Continued efforts to enhance its bioavailability and conduct rigorous clinical trials are essential to fully establish its clinical relevance in patient populations.

## 1. Introduction

Xanthohumol (XN) is a prenylated chalcone flavonoid derived from hops (*Humulus lupulus*), best known as a bioactive component of hops and beer. In recent years, XN has attracted broad research interest because it modulates multiple disease-relevant pathways involving inflammation, oxidative stress, metabolism, and cell survival. Rather than acting on a single molecular target, XN functions as a pleiotropic regulator, allowing it to influence a wide range of biological processes.

**Figure 1:**
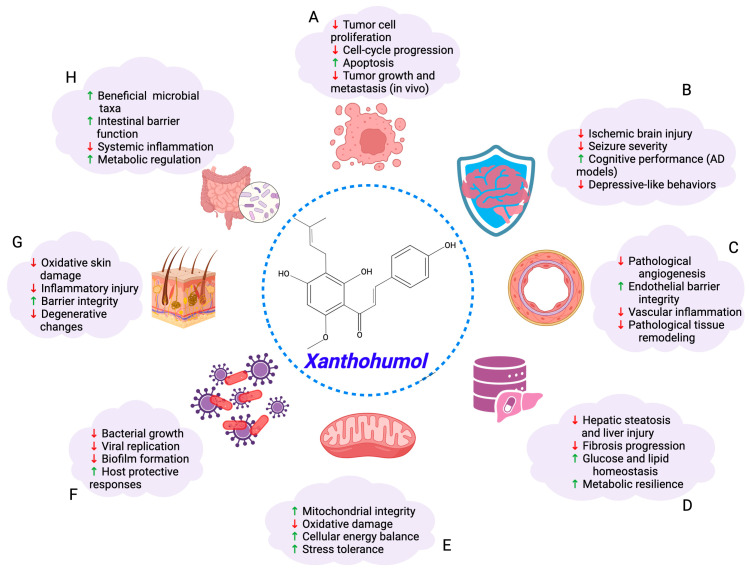
Broad biological effects of xanthohumol (XN) across disease-relevant systems. XN exerts pleiotropic biological effects across multiple organ systems and pathological contexts. (**A**) XN displays anticancer activity characterized by reduced tumor cell proliferation, induction of apoptosis, and suppression of tumor growth and metastasis. (**B**) In neurological settings, XN confers neuroprotection, including attenuation of ischemic injury, seizure severity, cognitive decline, and depressive-like behaviors. (**C**) XN also supports cardiovascular and endothelial protection by reducing pathological angiogenesis, limiting vascular inflammation, and preserving endothelial barrier integrity. (**D**) In hepatic and metabolic disorders, XN promotes hepatoprotection and metabolic regulation, reflected by reduced steatosis and fibrosis and improved glucose and lipid homeostasis. (**E**) Across multiple disease contexts, XN preserves mitochondrial integrity and redox balance, supporting cellular energy homeostasis and stress tolerance. (**F**) XN further exhibits broad antimicrobial and antiviral activity, including inhibition of bacterial growth, viral replication, and biofilm formation. (**G**) In skin, XN provides barrier-protective and anti-inflammatory effects, reducing oxidative damage and degenerative changes. (**H**) Emerging evidence also implicates gut microbiome modulation as a contributor to XN’s systemic effects, including improved barrier function and metabolic regulation. Collectively, these biological outcomes arise from coordinated modulation of conserved cellular stress-response networks, which are explored mechanistically in subsequent sections. Down arrow (red): downregulation; Up arrow (green): upregulation.

A growing body of research has demonstrated that XN possesses antioxidant, anti-inflammatory, antimicrobial, antiviral, and antitumor properties across diverse experimental systems. In cancer biology, XN preferentially targets malignant cells over matched normal counterparts, consistent with selective vulnerability to mitochondrial stress and cell cycle disruption. In addition to direct effects on tumor cells, XN influences the tumor microenvironment by suppressing angiogenesis and inflammatory signaling, thereby limiting tumor growth and progression (Figure 1).

Beyond oncology, XN exhibits antimicrobial and antiviral activity and confers protection in neurological, metabolic, cardiovascular, renal, hepatic, and dermatologic models. Across these contexts, XN preserves mitochondrial integrity, attenuates oxidative and inflammatory injury, and mitigates tissue damage associated with ischemia, metabolic stress, and chronic inflammation (Figure 1). Collectively, these observations support the view that XN acts on conserved cellular stress-response pathways that are relevant across multiple disease settings. Together, these observations reinforce the concept that XN influences conserved cellular stress-response pathways that are relevant across multiple disease settings.

Human studies demonstrate that oral XN is safe and well tolerated with observed broad therapeutic promises. However, its hydrophobic nature and rapid biotransformation pose challenges for systemic delivery. Ongoing formulation improvements, such as micellar encapsulation, have begun to enhance its bioavailability and translational potential [1,2,3,4,5,6,7,8,9,10,11,12,13].

In this review, a comprehensive literature search was conducted between 2000 and December 2025 using multiple scientific databases, including PubMed, Google Scholar, ScienceDirect, Wiley Online Library, and the MDPI database, to identify relevant studies on XN. The search strategy employed the keywords “xanthohumol” and “*Humulus lupulus*” in combination with disease- and mechanism-related terms, such as “cancer,” “neuroprotection,” “inflammation,” “oxidative stress,” “metabolism,” “AMPK,” “Nrf2,” and “NF-κB.” Inclusion criteria comprised full-text original research articles and human studies published in English that investigated the biological activity, molecular mechanisms, or safety of XN. Review articles were screened to identify additional relevant primary studies. Exclusion criteria included preprints, studies lacking compound characterization or purity information, and reports in which XN was not the primary bioactive compound discussed in the abstract. The publications identified through this process formed the core literature base for this integrative mechanistic and translational review.

In summary, current findings position XN as a versatile natural compound with potential applications spanning cancer therapy, neuroprotection, metabolic regulation and inflammatory disease management. Continued mechanistic and clinical investigations, including efforts to improve formulations, will be required to define its effective nutraceutical efficacy in the future.

**Figure 2:**
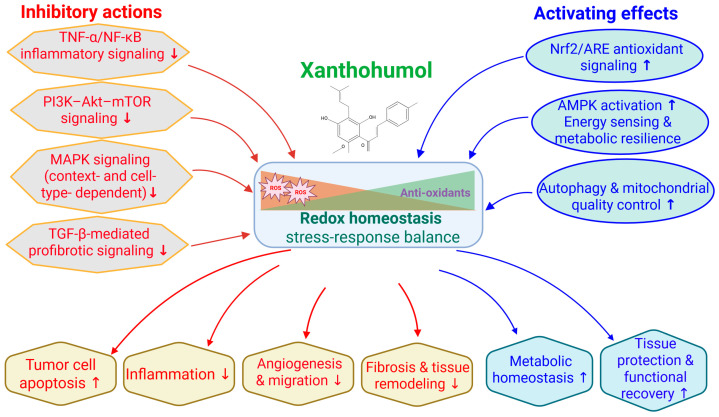
Integrated schematic illustrating the dual inhibitory and activating actions of xanthohumol (XN) on cellular signaling pathways, organized around a central redox stress-response balance. On the left (red color font and arrow flows), XN exerts inhibitory effects on pro-inflammatory and pro-pathogenic pathways, including TNF-α/NF-κB inflammatory signaling, PI3K–Akt–mTOR signaling, context- and cell-type–dependent MAPK signaling (ERK/JNK/p38), and TGF-β-mediated profibrotic signaling. On the right (blue color font and arrow flows), XN induces activating effects on cytoprotective pathways, including Nrf2/ARE-dependent antioxidant signaling, AMPK-mediated energy sensing and metabolic resilience, and autophagy-associated mitochondrial quality control. We posit that these opposing actions converge on a central regulatory node defined as redox homeostasis and cellular stress-response balance, reflecting the coordinated regulation of reactive oxygen species and antioxidant defenses. Downstream biological consequences of this rebalanced redox state include preferential induction of tumor cell apoptosis, suppression of inflammation, inhibition of angiogenesis and migration, attenuation of fibrosis and pathological tissue remodeling (red color font), improvement of metabolic homeostasis, and enhanced tissue protection and functional recovery (blue color font). Arrows indicate the direction of pathway modulation, highlighting mechanistic convergence rather than isolated pathway effects. Down arrow (red): downregulation; Up arrow (blue): upregulation.

Several sector-specific reviews have previously examined XN in individual disease contexts, including oncology, metabolic disorders, and cardiometabolic diseases. However, an integrated synthesis that reconciles these findings through shared mechanistic frameworks and translational relevance remains lacking. Accordingly, the present review does not aim to provide an exhaustive enumeration of all reported biological effects of XN. Instead, it adopts a mechanism-centered perspective, organizing evidence across disease areas according to convergent stress-response and metabolic signaling networks, with particular emphasis on redox modulation by inhibiting NF-κB and activating Nrf2 signaling axes (Figure 2).

By structuring the literature around these core regulatory nodes, this review highlights how apparently diverse outcomes—such as antiproliferative, anti-inflammatory, antiviral, neuroprotective, cardiometabolic, and hepatoprotective effects—arise from a limited set of interconnected upstream events rather than independent pathway-specific actions. Mechanistic details are therefore developed within the relevant disease sections, while translational considerations, including nutraceutical relevance, formulation, bioavailability, and human safety data, are integrated throughout. This approach is intended to bridge preclinical mechanistic insights with emerging human evidence and to clarify the potential positioning of XN at the interface of dietary bioactives and drug-like therapeutics.

## 2. Anticancer Effects

XN exhibits potent anticancer effects across diverse malignancies through multi-targeted mechanisms, including inhibition of NF-κB and AKT signaling, induction of mitochondrial dysfunction and apoptosis, and suppression of angiogenesis.

### 2.1. Cellular and In Vivo Anticancer Effects of XN

Antiproliferation: Concentration-dependent antiproliferative effects of XN have been consistently observed across multiple cancer cell types. Importantly, XN demonstrates notable selectivity for malignant cells over their normal counterparts. For example, the IC_50_ for normal CDD-18Co colon fibroblasts exceeded 100 µM, whereas the IC_50_ for HT-29 colon cancer cells was approximately 10 µM [14]. Sensitivity to XN is also influenced by the spatial architecture of the tissue model. In three-dimensional (3D) culture systems, cancer cells exhibited reduced susceptibility compared with two-dimensional (2D) monolayers: IC_50_ values for MCF-7 and A549 spheroids increased to 12.37 µM and 31.17 µM, respectively, compared with 1.9 µM and 4.74 µM in 2D cultures [15]. This difference underscores the importance of microenvironmental context in modulating XN responsiveness.

Cell cycle arrest and apoptotic induction: XN modulates key regulators of cell cycle progression, leading to arrest at various phases depending on the cancer type, with G_1_ and G_2_/M checkpoints most affected. G_1_ arrest is associated with reduced cyclin D1 expression [16] and upregulation of the cyclin-dependent kinase inhibitor p21 [8], whereas G_2_/M arrest correlates with depletion of cyclin B and increased phosphorylation of CDK1 (formerly cdc2) [14]. XN also elevates p53 levels [17], indicating activation of p53-dependent checkpoint pathways.

XN induces apoptosis across diverse malignancies through both intrinsic and extrinsic mechanisms. Hallmarks of apoptosis have been consistently reported, including nuclear fragmentation [14], DNA laddering [18], chromatin condensation [19], phosphatidylserine externalization [20], an increased ratio of Bax to Bcl-2 [14], activation of initiator caspases (caspase-8 and caspase-9) and executioner caspase-3 [18], loss of mitochondrial membrane potential [18], and release of cytochrome c [21].

Representative in vitro studies demonstrating the anticancer activity of XN across diverse tumor types, including colon, breast, lung, and hematologic malignancies, are summarized in Table 1.

In vivo tumor inhibition: XN has demonstrated robust antitumor activity across multiple animal- and patient-derived xenograft models, as summarized in Table 2. In SG-231 cholangiocarcinoma xenografts, XN treatment resulted in a fivefold reduction in tumor size by day 16 compared with untreated controls [22]. In a CCLP-1 model, XN suppressed tumor growth by 78%, whereas tumors in the control group expanded by 853% over the same period [22]. XN-treated tumors consistently showed a reduction in Ki-67 expression [23], along with an increase in central necrosis and apoptotic markers [24].

Anti-angiogenic effects: XN exhibits potent anti-angiogenic properties mediated largely through suppression of NF-κB-dependent VEGF and IL-8 expression. In breast cancer xenografts, oral XN administration reduced microvessel density and lowered numbers of endothelial cells, with NF-κB activity decreasing to approximately 60% of control values [24]. In pancreatic cancer cell lines (BxPC-3), XN inhibited VEGF and IL-8 production and blocked tube formation in human umbilical vein endothelial cells (HUVECs) [25]. Correspondingly, in BxPC-3 xenografts, XN significantly reduced CD31-positive microvessel density [25], confirming anti-angiogenic effects in vivo. Additional evidence comes from melanoma metastasis models, where XN-treated mice displayed enlarged areas of central necrosis within liver lesions, consistent with restricted blood supply [23]. Studies in multiple myeloma further showed decreased VEGF secretion following XN exposure [26], supporting its broad anti-angiogenic activity.

Anti-metastasis: XN inhibited multiple steps in the metastatic cascade, including cell migration, invasion, and adhesion to endothelial cells. XN suppressed leukemic cell invasion, metalloprotease production, and adhesion to endothelial cells, potentially preventing life-threatening complications of leukostasis and tissue infiltration [17]. In the acute lymphocytic leukemia model, administration of XN (2 mg/kg, assuming a 25g mouse) significantly prolonged animal lifespan by delaying neurological complications with leukemic dissemination [27]. XN also showed dose-dependent inhibition of melanoma cell migration in vitro and markedly reduced hepatic metastasis formation in vivo [23]. Mechanistically, studies from 3D cultures revealed that XN suppressed key invasion-related genes, including MMP2, MMP9, and FAK, accompanied by disruption of the actin microfilament network [15]. Collectively, these data support a multifaceted anti-metastatic role for XN across diverse tumor types.

### 2.2. Molecular Mechanisms of Action

Although XN has been reported to modulate multiple signaling pathways, such actions are not independent or in parallel. Instead, available evidence supports a hierarchical and interconnected mechanism in which a limited number of primary molecular events initiate broader downstream responses. The core of XN’s anticancer activity is direct redox-sensitive interactions between key regulatory proteins and mitochondria, leading to oxidative stress modulation and mitochondrial dysfunction. These primary events subsequently suppress central survival and inflammatory signaling nodes, most prominently the NF-κB and PI3K/AKT/mTOR pathways. Inhibition of these master regulators then drives secondary effects on angiogenesis, apoptosis, cell cycle progression, epithelial–mesenchymal transition, and metastatic behavior. Additional pathways, including MAPK and Notch signaling, appear to be context-dependent modulators that fine-tune cellular responses rather than universal primary targets. Framing XN’s actions within this mechanistic hierarchy helps reconcile its broad biological activity while emphasizing a coherent and biologically plausible mode of action (Figure 2).

NF-κB Pathway: XN directly targets key regulatory components of the NF-κB pathway by covalently modifying critical cysteine residues in IκB kinase (IKK) and p65, leading to inhibition of IKK activation, suppression of IκBα phosphorylation and degradation, and prevention of p65 nuclear translocation, thus decreasing NF-κB-dependent transcription [28]. Mutating these residues abolished XN’s effects [28], confirming their functional importance. XN demonstrated a 40% reduction in NF-κB activity in an MDA-MB-231 (Table 1) breast cancer xenograft [24] and inhibition of both constitutive and inducible NF-κB signaling in pancreatic cancer models [25]. Downstream consequences included reductions in anti-apoptotic proteins (survivin, Bcl-xL, XIAP, cIAP1, and cIAP2) [28], alongside decreases in pro-inflammatory cytokines (IL-1β) [24] and angiogenic factors (VEGF and IL-8) [25]. These combined effects contribute to XN’s ability to promote apoptosis, suppress inflammation, and impair tumor angiogenesis.

PI3K/AKT/mTOR Pathway: XN inhibits the PI3K/AKT/mTOR signaling cascade through direct and indirect mechanisms. In esophageal squamous cell carcinoma cell lines (KYSE70, KYSE450, and KYSE510; Table 1), which exhibit substantial biological heterogeneity, XN acted as an ATP-competitive inhibitor of AKT1/2, with computational docking models supporting direct binding to the kinase domain. This inhibition led to decreased phosphorylation of key downstream targets such as GSK3β, mTOR, and S6K, resulting in reduced cyclin D1 expression and G_1_-phase cell cycle arrest [29]. Similar findings were reported in gastric cancer models, where XN suppressed phosphorylation of PI3K, AKT, and mTOR in vivo [30]. In leukemia, XN-mediated downregulation of AKT signaling correlated with reduced cell viability and enhanced apoptosis [17]. These results collectively indicate that XN effectively disrupts PI3K/AKT/mTOR-driven survival and proliferation pathways across multiple tumor types.

Notch Signaling: XN exerts potent antitumor effects through inhibition of the Notch signaling pathway. In ovarian cancer cell lines (e.g., SKOV3 and OVCAR3), XN significantly suppressed cell growth by downregulating Notch1 transcription and protein expression, accompanied by increased Hes6 and decreased Hes1 transcription, with disrupted Notch signaling [31]. In pancreatic cancer models, XN reduced Notch promoter activity and downstream Notch effectors such as Hes-1, thus suppressing cell surviving. Enforced expression of constitutively active Notch1 reversed XN-mediated growth inhibition, confirming Notch1 as a direct functional target [32]. In cholangiocarcinoma cell lines (CCLP-1 and SG-231; Table 1), XN induced a time-dependent, stepwise reduction in Notch1 expression as early as 12 h after treatment, preceding later decreases in AKT phosphorylation [22]. This temporal sequence suggests mechanistic crosstalk in which Notch1 suppression contributes to downstream PI3K/AKT pathway attenuation. Collectively, these observations establish Notch1 inhibition as a key upstream event in XN’s anti-proliferative action across multiple tumor types.

MAPK Pathways: XN modulates multiple members of the MAPK family, producing context-dependent effects across cancer types. In HT-29 colon cancer cells (Table 1), XN induced a concentration-dependent blockade of the MEK/ERK, contributing to growth suppression [14]. In multiple myeloma, ERK and JNK activation were identified as essential mediators of XN-induced apoptosis; inhibition of these kinases or pretreatment with N-acetylcysteine prevented caspase-3 activation and apoptotic cell death [26]. In laryngeal cancer cells (Table 1), inhibition of ERK1/2 phosphorylation by XN was identified as a key mechanism underlying reduced cell proliferation [16]. In melanoma models, including B16-F10 cells (Table 1 and Table 2), XN inhibited JNK phosphorylation while modestly increasing p38 MAPK activation, indicating differential regulation of MAPK subfamilies [23]. In glioblastoma, XN activated the ERK/c-Fos axis, leading to upregulation of miR-204-3p, which subsequently suppressed the IGFBP2/AKT/Bcl-2 pathway [33]. Collectively, these findings indicate that XN targets multiple MAPK targets, with inhibitory or activating effects depending on the cellular context, ultimately converging on pathways that regulate growth arrest, apoptosis, and stress responses.

Mitochondrial Dysfunction and ROS Generation: XN-induced mitochondrial dysfunction through multiple mechanisms. A comprehensive mechanistic study in human cancer cell lines (Table 1) identified mitochondria as the primary site of superoxide anion generation following XN exposure, with EC_50_ values of XN 3.1 µM in human cancer cell lines and 11.4 µM in isolated mitochondria [34]. XN resulted in rapid ATP depletion (IC_50_ = 26.7 µM within 15 min), a 15% increase in oxidized glutathione, and dose-dependent thiol depletion (IC_50_ = 24.3 µM) [34]. The XN inhibited electron flux from respiratory chain complexes I and II to complex III, with IC_50_ values of 28.1 and 24.4 µM, respectively, leading to collapse of the mitochondrial membrane potential and release of cytochrome c [34]. Importantly, antioxidants such as N-acetylcysteine and MnTMPyP prevented XN-induced mitochondrial depolarization and apoptosis, confirming ROS generation as a key upstream trigger of XN’s cytotoxic activity [34]. Consistent with this mechanism, numerous studies have reported ROS production as a central component of XN’s anticancer effects [17].

Other Molecular Targets: Beyond its effects on the above major signaling pathways, XN interacts with several specific molecular targets. In esophageal squamous carcinoma cell lines, KYSE70, KYSE450, and KYSE510 (Table 1), XN was shown to bind keratin 18 and promote its degradation without altering mRNA expression [21]. In leukemia models, XN inhibited Bcr-Abl expression at both the mRNA and protein levels, contributing to reduced cell viability and enhanced apoptosis [17].

Synergistic Effects with Chemotherapy: XN has demonstrated strong chemosensitizing properties across multiple cancer models. In H1299 lung cancer cells (Table 1), XN enhanced cisplatin-induced DNA damage, with the combination producing greater γH2AX foci formation, a hallmark of DNA double-strand breaks, suggesting that XN could sensitize tumor cells to DNA-damaging chemonutraceuticals more than either agent alone [19]. XN at 6.25 or 12.5 µM in the H1299 lines enhanced cisplatin-induced apoptosis by activation of caspase-3 and cleavage of PARP-1 [19]. Drug-resistant breast cancer cell models (MDA-MB-231; Table 1) demonstrated that XN downregulated MDR1, EGFR, and STAT3, thereby restoring sensitivity to chemotherapeutic agents and radiation therapy [35]. Importantly, in acute lymphocytic leukemia, cells that adapted to long-term XN exposure became highly responsive to other cytotoxic drugs [27]. These findings highlight XN’s capacity to prime tumor cells for enhanced susceptibility to sequential or alternating treatment strategies.

XN also exhibits protection of normal DNA damage in response to chemotherapy. In a rat model, XN protected against genotoxic injury induced by the heterocyclic aromatic amine IQ, a dietary carcinogen [36]. Animals consuming XN-supplemented drinking water showed reductions in preneoplastic GST-p+ foci and DNA migration in both colon mucosae and liver cells [36]. Together, these findings highlight XN’s dual ability to protect normal tissues from carcinogen-induced DNA damage while potentiating DNA damage-based cancer therapies.

**Table 1:** In vitro anticancer effects of xanthohumol across tumor types.

Cancer Type	Cell Line (In Vitro)	Xanthohumol Concentration	Key Biological Outcomes	Primary Mechanistic Actions	Ref.
Colon	HT-29	IC_50_ = 10 µM	Growth inhibition; G_2_/M arrest	Cyclin B1 downregulation	[14]
Breast	MDA-MB-231	10–20 µM (2d)	Reduced survival; apoptosis induction	Intrinsic mitochondrial apoptosis (caspase-3/9 activation)	[18]
Breast (3D)	MCF-7	IC_50_: 1.9 µM (2D); 12.37 µM (3D)	Increased apoptosis and necrosis	Reduced sensitivity in 3D spheroids	[15]
Lung (3D)	A549	IC_50_: 4.74 µM (2D); 31.17 µM (3D)	Increased apoptosis and necrosis	Reduced sensitivity in 3D spheroids	[15]
Cholangiocarcinoma	CCLP-1	5–15 µM (2–4 d)	Time-dependent growth inhibition	G_0_/G_1_ arrest; caspase-3 activation; PARP cleavage	[22]
	SG-231	5–15 µM (2–4 d)	Time-dependent growth inhibition	G_0_/G_1_ arrest; caspase-3 activation; PARP cleavage	[22]
	CC-SW-1	5–15 µM (2–4 d)	Time-dependent growth inhibition	G_0_/G_1_ arrest; caspase-3 activation; PARP cleavage	[22]
Cervical	Ca Ski	IC_50_: 20–60 µM (2–3d)	Apoptosis induction; S-phase accumulation	S-phase cell cycle blockade	[20]
Esophageal	KYSE70/450/510	5 µM	70–80% growth inhibition; apoptosis	Cyclin D1 downregulation; G_1_ arrest	[29]
Ovarian	EOC lines	N.A.	S and G_2_/M arrest	Cell cycle checkpoint activation (p21, CDK1 modulation)	[31]
Thyroid	Human thyroid cells	N.A.	Cell cycle redistribution	G_1_ reduction; S-phase increase	[37]
Larynx	Larynx cancer cells	N.A.	Cell cycle arrest; apoptosis-related signaling	p53 and p21/WAF1 induction; Bcl-2 downregulation	[16]
Multiple myeloma	Myeloma cells	N.A.	Cell cycle blockade	Cyclin D1 downregulation; p21 induction	[26]
Lung	H1299	Xanthohumol ± cisplatin	Enhanced apoptosis vs. monotherapy	Chemosensitization via caspase-3 activation	[19]

IC_50_, half-maximal inhibitory concentration; PARP, poly(ADP-ribose) polymerase; CDK1, cyclin-dependent kinase 1; 2D, two-dimensional monolayer culture; 3D, three-dimensional spheroid culture; N.A., not available.

**Table 2:** In vivo anticancer effects of xanthohumol across tumor types.

Cancer Type	Animal Model/PDX System	Treatment (Dose, Route, Duration)	Primary Outcome	Mechanism Notes	Ref.
Breast	MCF7 xenograft (nude mice)	Oral XN; per study regimen	Central necrosis; ↓ inflammatory cells; ↑ apoptosis	Anti-angiogenic/anti-inflammatory context	[24]
Liver/colon (preneoplasia)	Rat model	71 µg/kg in drinking water	↓ GST-p+ foci 50%; ↓ foci area 44%	Suppression of preneoplastic lesions and DNA damage	[36]
Melanoma (metastasis)	B16-F10 (C57BL/6)	10 mg/kg i.p. pellets	↓ Hepatic metastasis & overall tumor load	Anti-metastatic effect	[23]
Cholangiocarcinoma	SG-231 xenograft (nude)	5 mg/kg i.p. qod ×16 days	Growth: 427%→153% (day 8); 1308%→255% (day 16)	Notch1/AKT inhibition	[22]
	CCLP-1 xenograft (nude)	5 mg/kg i.p. qod ×16 days	Growth: 381%→−13% (day 8); 853%→−78% (day 16)	Notch1/AKT inhibition	[22]
Pancreatic	BxPC-3 xenograft	Weekly i.p. injections	↓ Tumor volume (significant)	NF-κB suppression	[25]
Gastric	SGC-7901 xenograft	0.5–1 mg/kg, i.p., once daily × 3 weeks	↓ Tumor volume & weight	PI3K/Akt/mTOR inhibition	[30]
Esophageal	KYSE30 PDX (SCID mice)	40–160 mg/kg OG, daily ×64 d	Significant tumor growth decrease	AKT/MAPK axis modulation	[21]
	High-AKT PDX(SCID mice)	80–160 mg/kg OG, daily ×50 d	↓ Tumor volume & weight (greater in high-AKT)	AKT dependency noted	[29]

PDX, patient-derived xenograft; i.p., intraperitoneal; qod, every other day; GST-p^+^, glutathione S-transferase placental form; AKT, protein kinase B; OG, oral gavage. Down arrow: decrease; Up arrow: increase.

## 3. Antiviral and Antimicrobial Effects

XN exhibits broad-spectrum antimicrobial activity, demonstrating potent effects against drug-resistant bacteria, coronaviruses, hepatitis C virus (HCV), HIV-1, parasites, and fungi. Its mechanisms include membrane disruption, inhibition of viral protease, and interference with key replication pathways. In addition, reported therapeutic indices indicate a favorable safety margin, underscoring XN’s potential as a multifunctional antimicrobial agent.

### 3.1. Antimicrobial and Antiviral Activity of XN

Antimicrobial effects: XN demonstrates potent activity against Gram-positive bacteria, including clinically significant drug-resistant strains. XN at 84.3% purity produced marked reductions in *Clostridioides difficile* (strain 176) by day 3 in a rat infection model, representing the strongest antimicrobial effect among the hop-derived compounds [38]. Purified XN exhibited MIC values ranging from 15 to 107 μg/mL against toxigenic *C. difficile* isolates, with activity approaching that of standard antibiotics even in resistant strains [39], demonstrating XN’s potential as a promising candidate for managing difficult-to-treat Gram-positive infections.

Against *Staphylococcus aureus*, pure XN exhibited exceptional potency, achieving an MIC of 3.9 μg/mL against MRSA ATCC 43300—classified as very strong antimicrobial activity by established criteria [40]. Remarkably, XN maintained comparable efficacy against both methicillin-resistant and methicillin-sensitive strains, with MIC values around 4 mg/L for *staphylococci* and complete bacterial reduction at approximately 60 μg/mL in biofilm assays [40,41]. In biofilm eradication studies, XN at the MIC concentration reduced biofilm viability by 86.5%, substantially outperforming spent hop extract (42.8%), and achieved near-complete eradication (97–99%) at higher doses [41]. XN also displayed meaningful activity against *enterococci*, with MIC values of 7.5 mg/L and efficacy extending to vancomycin-resistant *E. faecium* [40].

Representative antibacterial, antiparasitic, and antifungal activities of XN across diverse pathogens, including Gram-positive bacteria, biofilm-forming communities, parasites, and fungi, are summarized in Table 3.

Several mechanisms underlie XN’s antimicrobial effects. XN disrupts cell membrane integrity, interferes with fatty acid metabolism, and promotes intracellular proton accumulation leading to metabolic starvation [38,39]. Its hydrophobic structure facilitates penetration of bacterial cell walls and incorporation into inner membranes, where XN functions as a mobile carrier ionophore. This activity drives electroneutral proton influx, inhibits active transport systems, and ultimately limits the uptake of essential sugars and amino acids [39].

Anti-biofilm: In multispecies biofilm models designed to simulate oral peri-implant conditions, XN at 100 μM exhibited striking antimicrobial activity against six bacterial species associated with peri-implantitis, showing inhibition rates ranging from 95.90% to 99.20% [42]. XN-treated biofilms displayed markedly reduced biomass and cell viability and, in several cases, achieved greater reductions than chlorhexidine. Structural disruption of the biofilms included architectural degradation. These findings highlight XN’s strong potential as an anti-biofilm agent in oral infectious disease settings [42].

Mechanistically, XN exerts activity against biofilm-forming bacteria by targeting lipid metabolism, resulting in altered cell wall hydrophobicity and impaired adhesion. XN also disrupts quorum-sensing pathways essential for microbial communication and coordinated biofilm formation [43]. XN acts as a diacylglycerol acyltransferase inhibitor [43], destabilizing bacterial membranes by interfering with lipid synthesis and cell wall integrity. Together, these mechanisms position XN as a multifaceted antimicrobial agent capable of targeting both planktonic and biofilm-embedded pathogens.

Anti-coronavirus: XN exhibits potent antiviral activity across multiple coronaviruses, as summarized in Table 4, through direct inhibition of the viral main protease (Mpro), a pivotal enzyme required for viral replication [44]. Pretreatment with XN restricted viral replication in Vero-E6 cells [44]. XN achieved an IC_50_ of 1.53 μM against SARS-CoV-2 and an IC_50_ of 7.51 μM for alphacoronavirus PEDV [44]. XN displays strong antiviral effects against porcine reproductive and respiratory syndrome virus (PRRSV). Treatment with XN (5–15 μM) produced dose-dependent decreases in virus titers and viral mRNA levels with a selectivity index greater than 10 [45]. XN blocked PRRSV entry and internalization into cells and exerted its antiviral effects partially through activation of the Nrf2-HMOX1 antioxidant pathway, leading to increased expression of Nrf2, HMOX1, GCLC, GCLM, and NQO1, a dual mechanism blocking viral entry and enhancing cellular antioxidant defenses [45].

Anti-HCV: XN demonstrated dose-dependent activity of anti-HCV replicons [46]. At concentrations of 7.05–14.11 μM, XN achieved inhibitory effects comparable to interferon-alpha 2b, producing similar reductions in HCV RNA levels [46]. In the bovine viral diarrhea virus (BVDV) surrogate model of HCV, XN exhibited EC 50 values of 2.77–3.24 mg/L, with therapeutic indices exceeding 7.72–9.03 [47]. XN suppressed BVDV E2 expression and viral RNA in a dose-dependent manner. Comparatively, XN showed stronger inhibitory activity than ribavirin but was less potent than interferon-alpha [47]. In vivo, using the *Tupaia belangeri* model infected with HCV-positive serum, XN significantly reduced serum aminotransferases, histological activity scores, hepatic steatosis, and hepatic TGF-β1 expression, demonstrating both antiviral and hepatoprotective effects [48].

Anti-HIV-1, anti-HSV, and other viruses: XN exhibits anti-HIV-1 activity. In C8166 lymphocytes, XN achieved a therapeutic index of approximately 10.8 by suppressing virus-induced cytopathic effects, with an EC50 value of 0.82 μg/mL, reducing viral p24 antigen production at 1.28 μg/mL and inhibiting reverse transcriptase activity at 0.50 μg/mL [49]. In peripheral blood mononuclear cells (PBMCs), the EC50 was higher (20.74 μg/mL), reflecting cell-type-dependent sensitivity. Notably, XN did not inhibit recombinant HIV-1 reverse transcriptase activity or viral entry directly [49], indicating that its antiviral effects may involve alternative host- or virus-directed mechanisms that remain to be elucidated.

A XN-enriched hop extract (with >99% purity of XN) demonstrated low-to-moderate antiviral activity against multiple DNA and RNA viruses [50]. XN was more potent than isoxanthohumol against HSV-1 and HSV-2, with therapeutic indices of >1.9 and >5.3 respectively. Conversely, isoxanthohumol showed superior activity against CMV. XN exhibited a therapeutic index of 4.0 against rhinovirus [50].

XN also shows promise against arboviruses. For Oropouche virus (OROV), a member of *Peribunyaviridae*, XN demonstrated inhibitory activity with an EC50 of 50.2 μg/mL and a selectivity index of 4.9 [51]. Computational docking and biochemical analyses identified interactions between XN and residues Lys92 and Arg33 of the OROV endonuclease domain, an essential component of cap-snatching viral transcription [51].

Antiparasitic and antifungal effects: XN demonstrates substantial antiparasitic activity across multiple species [52]. XN exhibited the strongest antiplasmodial effect among eight tested chalcone derivatives, with IC_50_ values of 8.2 ± 0.3 μM for the chloroquine-sensitive poW strain and 24.0 ± 0.8 μM for the multidrug-resistant Dd2 clone [52]. Several chalcones, including XN, were shown to interfere with glutathione-dependent hemin degradation, a vital metabolic process for *P. falciparum* survival [52].

XN inhibits *Eimeria* species associated with coccidiosis. At 22 ppm, XN reduced *E. tenella* sporozoite invasion of MDBK cells by 66%, with physical disruption of parasite apical structures [53]. In vivo chick models demonstrated that pretreatment of *E. tenella* and *E. acervulina* sporozoites with 5–20 ppm XN reduced gross lesion scores and resulted in either normal weight gains or reduced oocyst shedding [53]. XN displayed inhibitory activity against *Babesia microti* with an IC_50_ of 21.40 μM and a selectivity index > 4.7 [54]. XN (50 μM) reduced parasitemia in infected erythrocytes to 2.07%, exhibiting similar effects of diminazene aceturate [54].

XN exhibits broad antifungal activity against *Candida albicans*, such as *C. krusei*, *C. tropicalis*, and *C. parapsilosis* [55]. In multispecies biofilm models, XN inhibited filamentous growth of *C. albicans* and partially reversed the disruptive effects of fungal overgrowth on periodontopathogenic bacteria. XN suppressed biomass and cell viability in biofilms [42,55].

### 3.2. Selectivity and Safety Profile

The therapeutic windows of XN varied across pathogens and study designs. For example, the therapeutic index was about 10.8 against HIV-1 [49], 7.72–9.03 against BVDV as an HCV surrogate [47], >5.3 against HSV-2, 4.0 against rhinovirus [50], >10 against PRRSV [45], and about 4.9 for OROV [51]. A comparative overview of antiviral selectivity indices and nutraceutical windows is provided in Table 4.

Hop derivatives demonstrated low cytotoxicity and low absorption [39]. In tuberculosis studies, oral administration of XN at various doses proved safe, with no significant changes in biochemical parameters or liver indices compared to control groups [56]. One notable safety consideration emerged in biofilm studies, where XN may have exhibited cytotoxic effects at concentrations above 100 μM [42,55], although such levels are typically higher than those required for antimicrobial or antiparasitic activity. Importantly, XN has shown hepatoprotective properties when co-administered with isoniazid during tuberculosis treatment. The combination reduced drug-induced liver injury, as evidenced by decreases in ALT, AST, ALP, bilirubin, and MDA, along with increases in SOD, GSH-Px, and ATPases. This protective effect operated through activation of antioxidative defense systems and protection of hepatocellular membranes [56]. Together, these findings indicate that XN possesses a favorable safety profile and may provide added protective benefits in combination therapeutic settings.

### 3.3. Comparative Effectiveness with Standard Treatments

XN frequently demonstrates antimicrobial and antiviral potency comparable to, or exceeding, several standard treatments. Against *C. difficile* and *Staphylococcus aureus*, XN’s MIC/MBC values approach those of conventional antibiotics. XN also outperformed other hop-derived compounds, including lupulone, humulone [40], α- and β-bitter acids, and commercial extracts [39], across multiple studies. Methanolic extracts enriched in XN were markedly more active than essential oils, and antimicrobial performance correlated strongly with XN content rather than α-acid concentration [57]. Synergistic interactions were observed when XN was combined with standard antibiotics, enhancing the activity of oxacillin against MSSA and linezolid against both MSSA and MRSA [41]. In tuberculosis models, XN combined with isoniazid produced the lowest lung and spleen colony-forming unit counts compared to all other treatment groups [56]. XN plus isoniazid outperformed isoniazid monotherapy in both antibacterial efficacy and hepatoprotective activity [56].

In biofilm models, XN showed a mixed but notable performance relative to chlorhexidine, achieving higher reductions for some bacterial species and performing slightly below curcumin for most [42,55]. Pure XN exhibited strong biofilm-eradicating capacity, reducing viability by 86.5% at MIC and approaching near-complete eradication at higher concentrations [41]. However, in staphylococcal biofilm assays, lupulone showed the strongest effect at high concentrations, followed closely by XN, with both achieving complete eradication at elevated doses [41].

Relative to antiviral standards, XN achieved inhibitory effects comparable to interferon-α2b in HCV replicon systems and displayed greater potency than ribavirin but less than interferon-α in the BVDV surrogate model [46,47]. Synergy with interferon-α has also been observed against HCV. A combination of 3.13 μg/mL XN with 50 IU/mL IFN-α yielded greater inhibitory effects on viral RNA levels than either agent alone at higher concentrations (6.25 μg/mL XN or 100 IU/mL IFN-α) [58].

**Table 3:** Antimicrobial and antifungal effects of xanthohumol.

Pathogen Group	Species/Strain (Model)	Concentration	Mechanism Highlights	Ref.
Oral biofilm (Gram+)	*S. oralis*, *A. naeslundii*, *V. parvula*	100 µM	Biofilm viability/biomass ↓	[42]
Oral biofilm (Gram− mix)	*F. nucleatum*, *P. gingivalis*, *A. actinomycetemcomitans*	100 µM	Biofilm viability/biomass ↓	[42]
Oral biofilm (mixed)	*F. nucleatum*, *P. gingivalis*, *A. actinomycetemcomitans*	N.A.	Candida–bacteria community structure normalized	[55]
Parasite (malaria, CQ-sens.)	*P. falciparum* (poW)	IC_50_ = 8.2 ± 0.3 µM	Interference with hemin detox	[52]
Parasite (malaria, MDR)	*P. falciparum* (Dd2)	IC_50_ = 24.0 ± 0.8 µM	Interference with hemin detox	[52]
Parasite (coccidia)	*E. tenella* (MDBK invasion)	22 ppm	Physical disruption of apical ends	[53]
Parasite (coccidia)	*E. acervulina*	5–20 ppm	Anti-invasion effects	[53]
Parasite (babesiosis)	*Babesia microti*	IC_50_ = 21.40 µM	Likely mitochondrial/ROS modulation	[54]
Fungi (Candida spp.)	*C. albicans*, *C. krusei*, *C. tropicalis*, *C. parapsilosis*	N.A.	N.A.	[40]
Fungi–bacteria biofilm	*C. albicans* within mixed biofilm	N.A.	Biofilm structure & vitality normalized	[55]

IC_50_, half-maximal inhibitory concentration; CQ, chloroquine; ROS, reactive oxygen species; MDBK, Madin–Darby bovine kidney cells; N.A., not available. Down arrow: decrease.

**Table 4:** Antiviral effects of xanthohumol.

Virus	System (Cell/Animal)	XN Dose/EC50/IC_50_	Selectivity Index	Primary Readouts	Ref.
SARS-CoV-2	Vero-E6; Mpro enzyme	IC_50_ (Mpro) = 1.53 µM	N.A.	Replication restricted in Vero-E6	[44]
PEDV (alpha-CoV)	Cell culture	IC_50_ = 7.51 µM	N.A.	Replication restricted	[44]
PRRSV (BB0907, S1, FJ1402)	Marc-145 & porcine alveolar macrophages	5–15 µM	SI > 10	Dose-dependent ↓ titers and mRNA	[45]
HCV replicon	Huh7.5 replicon	3.53–14.11 µM	N.A.	Luciferase & RNA ↓; effects ≈ IFN-α at 7.05–14.11 µM	[46]
HCV surrogate (BVDV)	MDBK	EC_50_ = 2.77–3.24 mg/L	TI > 7.72–9.03	CPE, E2, RNA ↓ (2.88–3.83 log10)	[47]
HCV surrogate (BVDV)	MDBK	3.13 µg/mL XN + 50 IU/mL IFN-α	N.A.	Viral RNA ↓ greater than either alone	[58]
HCV in vivo	*Tupaia* model (HCV-positive serum)	N.A.	N.A.	↓ aminotransferases, steatosis, histological activity index	[48]
HIV-1	C8166; PBMCs	EC50 = 0.50–1.28 µg/mL (C8166); 20.74 µg/mL (PBMCs)	~10.8 (C8166)	p24 & RT production ↓	[49]
Broad panel (BVDV, HSV-1/2, CMV, RV)	Cell culture	Low µg/mL range	HSV-2 TI > 5.3; RV TI = 4.0; CMV TI ≈ 4.2 (iso-α-acids)	CPE ↓	[50]
Oropouche virus (OROV)	Cell culture & in silico analysis	EC50 = 50.2 µg/mL	SI = 4.9	Replication ↓	[51]

IC_50_, half-maximal inhibitory concentration; EC_50_, half-maximal effective concentration; SI, selectivity index; TI, therapeutic index; IFN-α, interferon alpha; CPE, cytopathic effect; PBMCs, peripheral blood mononuclear cells; MDBK, Madin–Darby bovine kidney cells; RV, rhinovirus; CMV, cytomegalovirus; HSV, herpes simplex virus; Mpro, main viral protease; N.A., not available. Down arrow: decrease.

## 4. Neuroprotective and Neuromodulatory Effects

XN demonstrates broad neuroprotective effects across acute neurological injuries, chronic neurodegenerative diseases, and psychiatric conditions. These benefits arise through multiple complementary mechanisms, including Nrf2-mediated antioxidant defenses, inhibition of NF-κB signaling, prevention of apoptosis, enhancement of autophagy, and modulation of gut–brain axis pathways.

### 4.1. Neuroprotective Effects Across Neurological Conditions

Acute Neuroprotection: In middle cerebral artery occlusion-mediated cerebral ischemia models, administration of XN (0.2 or 0.4 mg/kg) 10 min before injury produced dose-dependent neuroprotection, exhibiting a reduction in infarct volume and improvement of neurological function [59]. These benefits were accompanied by suppressed expression of inflammatory proteins, including hypoxia-inducible factor-1α, tumor necrosis factor-α, phosphorylation of p38, inducible nitric oxide synthase, and active caspase-3 [59,60]. A comparative summary of neurological condition-specific models, XN regimens, functional outcomes, and associated biomarker changes is provided in Table 5.

In intracerebral hemorrhage, XN improved neurologic scores and reduced brain edema at 24 h post-injury. It attenuated neuronal apoptosis and decreased expression of pro-inflammatory mediators; effects were mediated through suppression of p65 phosphorylation in brain tissue [61]. In kainic acid-induced excitotoxic injury, pretreatment with XN (10 or 50 mg/kg) markedly reduced seizure severity, prevented excessive glutamate elevation, and protected CA3 hippocampal neurons. Mechanistically, XN restored levels of the mitochondrial fusion protein Mfn-2 and the anti-apoptotic protein Bcl-2, while inhibiting Apaf-1 expression and caspase-3 activation [62].

XN also showed protection against light-induced retinal degeneration. XN with doses of 0.4 and 0.8 mg/kg preserved visual acuity by approximately 50% and maintained 30% to 90% of photoreceptor function [63]. Histological analyses showed preservation of outer nuclear layer cell counts and reductions in apoptotic cells. XN further stabilized glutathione disulfide and cystine redox potentials, supporting its role in antioxidant defense [63].

Alzheimer’s Disease (AD) Models: XN demonstrates broad and consistent neuroprotective activity across multiple AD cellular and animal models. In N2a/APP cells, XN at 0.75–3.0 μM reduced accumulation of Aβ_1–42_ and Aβ_1–40_ and lowered their ratio. It also ameliorated tau hyperphosphorylation at Ser404, Ser396, and Ser262 through modulation of PP2A and GSK3β signaling. Proteomic analysis identified 30 differentially expressed proteins affected by XN, spanning pathways involved in ER stress, oxidative stress, proteasome function, and cytoskeletal organization [64].

In APP/PS1 transgenic mice, XN treatment for two months improved spatial learning and memory, evidenced by reduced latency and increased time spent in the Morris water maze target quadrant [65]. These behavioral benefits corresponded to reduced hippocampal Aβ deposition, increased superoxide dismutase levels, and decreased IL-6 and IL-1β in both serum and the hippocampus. Mechanistic analyses showed activation of autophagy (mTOR/LC3) and anti-apoptotic (Bax/Bcl-2) pathways [65]. XN enhanced ATP synthesis and mitophagy in the young AD hippocampus [66].

Dose–response differences were observed in another APP/PS1 study: XN (0.5 mg/kg) altered 108 hippocampal proteins, whereas a dose of 5 mg/kg altered only 72, suggesting greater responses at lower doses [67]. XN improved spatial learning and memory, enhanced newborn neurons in the subgranular zone and dentate gyrus, and decreased inflammatory responses [68]. Importantly, XN (5 mg/kg every other day for 90 days) reshaped gut microbiota in both prevention (2-month-old) and therapeutic (6-month-old) settings, modulating taxa such as Gammaproteobacteria, Nodosilineaceae, and Rikenellaceae, and influencing metabolic pathways related to penicillin/cephalosporin biosynthesis and atrazine degradation [68,69].

XN improved AD-related metabolic and cognitive outcomes in high-fat diet models. Dietary XN (0.07%, ~60 mg/kg/day) elicited sex- and ApoE-dependent improvements in learning and memory and increased hippocampal and cortical expression of glucose transporters, with elevation of Glut1 and Glut3. XN elevated diacylglycerol and sphingomyelin levels in females but decreased them in males, with lipid signatures correlating with improved cognitive performance [70,71].

Depression and Stress-Related Disorders: XN exhibits antidepressant and neuroprotective effects in models of depression and stress-induced neurological dysfunction. In the chronic unpredictable mild stress model, XN (20 mg/kg) alleviated depressive-like behaviors and increased synaptic protein expression in the medial prefrontal cortex. Treatment also reduced pro-inflammatory cytokines and oxidative stress [72]. XN also protected primary cortical neurons from corticosterone-induced cytotoxicity in stress-like disorders by preventing cell viability loss and preserving neuronal and astrocytic populations [73]. It restored brain-derived neurotrophic factor (*Bdnf*) mRNA levels, and its neuroprotective effects were abolished by Nrf2 inhibition, highlighting Nrf2 as a critical mediator specific to XN, in contrast to quercetin, which modulated Fkbp5 [73].

Pain Syndromes: XN demonstrates analgesic and anti-inflammatory efficacy in models of neuropathic and inflammatory pain [74]. In chronic constriction injury-induced neuropathic pain, XN reversed reductions in thermal withdrawal latency and mechanical withdrawal thresholds [75]. These behavioral improvements were accompanied by suppressed spinal cord production of pro-inflammatory cytokines TNF-α and IL-1β, along with reduced phosphorylation of ERK and NF-κB p65 [75].

In collagen-induced arthritis, XN administered intraperitoneally for three days reduced spontaneous pain behaviors, increased mechanical pain thresholds, and prolonged withdrawal latency [75]. Mechanistically, XN diminished the NLRP3 inflammasome in the spinal cord, enhanced Nrf2-dependent antioxidant responses, and lowered mitochondrial ROS production. Structural and biochemical analyses further demonstrated that XN binds to AMPK via two electrovalent bonds and increases its phosphorylation at Thr174, identifying AMPK activation as a key contributor to XN’s analgesic effects [75].

Other Neurological Conditions: XN demonstrates neuroprotective and cognition-enhancing effects across several neurological and aging-related models. In ovariectomy-induced cognitive decline models, XN reversed deficits in the Morris water maze and open field tests. Mechanistically, XN suppressed the injury-associated increase in miR-532-3p and restored expression of its downstream target *Mpped1* in the hippocampus. Functional studies confirmed that the 3′UTR of *Mpped1* is directly regulated by miR-532-3p. Overexpression of Mpped1 in the hippocampus relieved cognitive impairment, demonstrating a causal relationship [76].

In iron overload-induced nerve injury, both hops extract and XN improved memory performance, reflected by shortened escape latency, increased platform crossings, and improved spontaneous alternation ratios. XN treatment elevated hippocampal antioxidant enzymes (SOD and GSH-Px) and lowered lipid peroxidation markers (MDA), while reducing ROS levels in PC12 cells exposed to iron dextran [77].

In an epilepsy model, pretreatment with oral XN (20 mg/kg) reduced pentylenetetrazol (PTZ)-induced seizure onset and duration, mortality, and behavioral abnormalities [78]. XN decreased neuroinflammatory mediators (COX-2, TNF-α, NF-κB, TLR-4, and IL-1β) and oxidative stress markers (MDA and NO), while increasing GSH and SOD (*p* < 0.05). It also lowered glutamate levels and improved dopamine, GABA, Na^+^/K^+^-ATPase, and Ca^2+^-ATPase activities. Histopathology confirmed reduced inflammation and neuronal pyknosis [78].

In aging SAMP8 mice, chronic XN treatment reduced brain levels of IL-2, TNF-α, and IL-6, and blunted age-related increases in TNF-α, IL-1β, HO-1, iNOS, and GFAP expression [79]. XN counteracted synaptic decline by restoring mature BDNF and reducing pro-BDNF levels and increased the synaptic markers synapsin-I and synaptophysin [79].

### 4.2. Molecular Mechanisms of Neuroprotection

XN exerts neuroprotection through multiple convergent mechanisms that vary across neurological conditions but consistently involve antioxidant, anti-inflammatory, anti-apoptotic, synaptic, metabolic, and regulatory pathways. The major neuroprotective pathways engaged by XN in neural systems, together with linked functional outcomes and behavioral assays, are summarized in Table 6.

Nrf2-mediated antioxidant defense (core mechanism): XN robustly activates the Nrf2 pathway across models of depression, pain, ischemia, excitotoxicity, and iron overload [59,77]. It increases expression of HO-1, NQO1, SOD, and related antioxidant enzymes; reduces mitochondrial ROS; and prevents oxidative–nitrosative stress. Causal evidence comes from studies where Nrf2 inhibition blocks XN-mediated protection [73]. Consistent Nrf2 engagement across depression, retina, and oxidative injury paradigms is reflected in Table 6.

Anti-inflammatory signaling: XN suppresses several inflammatory cascades, including the NF-κB, NLRP3, p38-MAPK, ERK, and TLR4 pathways [59,61,72,75]. This results in broad reductions in pro-inflammatory cytokines such as TNF-α, IL-1β, and IL-6 across multiple disease models [65,75].

Anti-apoptotic and autophagic regulation: XN modulates Bax/Bcl-2 ratios, activates mTOR/LC3-dependent autophagy, and prevents caspase activation. In excitotoxicity and AD models, XN preserves mitochondrial integrity through upregulation of Mfn-2 and Bcl-2 and suppression of Apaf-1 and cleaved caspase-3 [62,65].

Tau and kinase pathway regulation: In AD models, XN reduces tau hyperphosphorylation by regulating PP2A and GSK3β signaling [64]. In iron overload models, XN activates AKT/GSK3β, further supporting neuroprotection [77].

Synaptic and neurotransmission modulation: XN normalizes excessive postsynaptic glutamate receptor expression in APP/PS1 mice [68]. In synaptosomes, it inhibits 4-aminopyridine-evoked glutamate release by reducing Ca^2+^ influx through N- and P/Q-type channels and suppressing Ca^2+^/calmodulin-PKA signaling via GABAa receptor engagement [80].

Energy metabolism and AMPK activation: XN improves metabolic resilience by activating AMPK (via direct binding and Thr174 phosphorylation) [75], enhancing ATP synthesis and mitophagy [68], and increasing Glut1/Glut3 expression in the hippocampus and cortex [70,71].

Gut–brain axis modulation: XN reshapes gut microbiota composition, modulates taxa, and alters microbial metabolic pathways in both preventive and therapeutic AD models [68,69]. It also correlates gut microbiome changes with hippocampal proteomics [67].

miRNA regulation: XN regulates disease-associated miRNAs, for example, suppressing miR-532-3p and restoring its target Mpped1 to improve cognition in ovariectomy-induced decline [76].

Adenosine pathway modulation: XN increases adenosine A1 receptor levels and decreases CD73 activity [81], potentially reducing excitotoxicity in conjunction with its glutamate-modulatory effects [62,80].

### 4.3. Translational Considerations and Study Limitations

Across neurological models, XN displayed clear dose-dependent neuroprotective patterns. Timing was a critical determinant of efficacy. Pre-injury dosing produced the most consistent protection: 10 min before ischemia [59], 30 min before excitotoxic seizures [62], and 30 min before PTZ-induced epilepsy [78]. For degenerative processes, chronic administration was essential, with benefits emerging after weeks to months of treatment. The diversity of dosing regimens and temporal windows across neurological models is summarized in Table 5.

Several studies identified limitations relevant to advancing XN toward clinical use. Bioavailability and delivery challenges were repeatedly noted. Retinal degeneration studies emphasized that effective neuroprotective concentrations cannot be achieved through dietary (beer) consumption and require dedicated pharmacokinetic and pharmacodynamic optimization [63]. In microbiome-modulating AD studies, oral absorption of XN may have been suboptimal, potentially reducing effect sizes [69], and treatment durations may not have been long enough to capture late-stage cognitive decline in APP/PS1 mice [68]. Several studies acknowledged important limitations and translational challenges. In light-induced retinal degeneration studies, pharmacokinetic and pharmacodynamic studies were identified as necessary to understand how XN is transported into the retina. The authors noted that achieving neuroprotective plasma concentrations through beer consumption is unlikely, highlighting the need for alternative delivery methods. Further experiments were needed to verify the mechanism of XN’s antioxidant response [63].

The limitations related to methodologies and model systems included: reliance on behavioral readouts without neuropathological confirmation [68], limited resolution of 16S rDNA sequencing for microbiome analysis [69], and the inherent reductionism of in vitro AD models [64]. High-fat diet studies revealed ApoE isoform- and sex-specific responses, limiting generalizability and emphasizing the need to model diverse populations [70,71]. Age also influenced responsiveness, with reduced efficacy observed in older animals [71]. In corticosterone-induced cytotoxicity, gaps remain in understanding polyphenol neuroprotection pathways [73]. Age-related cognitive studies also revealed confounds such as poor performance in young mice due to phytoestrogen-deficient diets and limited efficacy in older animals [82]. In adenosine pathway studies, discrepancies between gene and protein expression suggested methodological or timing-related limitations [81].

**Table 5:** Neuroprotective effects of xanthohumol.

Condition/Model	Species/Cell Line	Xanthohumol Regimen	Key Functional Outcomes	Key Biomarkers/Readouts	Refs.
Cerebral ischemia (MCAO)	Rat MCAO	0.2–0.4 mg/kg; −10 min pre-occlusion	↓ infarct size; improved neuro scores	↓ HIF-1α, TNF-α, iNOS; ↓ active caspase-3; ↓ p-P38 and ↑ Nrf2	[59,60]
Intracerebral hemorrhage	Rat ICH	Peri-injury dosing (per paper)	↑ neurologic scores; ↓ brain edema	↓ p65-P; ↓ apoptosis; ↓ inflammatory mediators	[61]
Excitotoxicity (kainate)	Rat (KA)	10 or 50 mg/kg, −30 min	Seizures ameliorated; CA3 neuron protection	↑ Mfn-2, Bcl-2; ↓ Apaf-1, cleaved caspase-3; ↓ glutamate	[62]
Retinal degeneration (light-induced)	Mouse	0.4–0.8 mg/kg; −1 d, −1 h, then q3d	~50% VA preserved; 30–90% PR function retained	↓ TUNEL+; ONL nuclei preserved; redox homeostasis	[63]
AD cell model (N2a/APPswe)	Neuro2a/APPswe	0.75–3.0 µM	↓ Aβ1-42/1-40; ↓ Aβ ratio	↓ tau pS404/pS396/pS262 via PP2A & GSK3β	[64]
AD transgenic (APP/PS1)	Mouse APP/PS1	30–90 mg/kg/day, oral gavage, 6 d/week × 2 months	MWM: ↓ latency; ↑ target time	↓ hippocampal Aβ; ↑ SOD; ↓ IL-6/IL-1β; ↑ mTOR/LC3; Bax/Bcl-2 shift	[65]
AD transgenic (microbiome–therapy/prevention)	Mouse APP/PS1	5 mg/kg qod ×90 d	Cognitive protection; prevention vs. therapy differences	ATP↑; mitophagy↑; blood/intestine glutamate↓	[69]
AD transgenic (dose–proteome)	Mouse APP/PS1	0.5 vs. 5 mg/kg	0.5 mg/kg: 108 proteins altered vs. 72 at 5 mg/kg	Broader hippocampal proteome shift at lower dose	[67]
Diet-induced cognitive impairment	WT & FXR intestine-KO mice; ApoE stratified	0.07% diet (~60 mg/kg/d), 10–19 weeks	Learning/memory improved (sex- & ApoE isoform-dependent)	↑ Glut1, ↑ Glut3; lipidome shifts	[71]
Chronic stress-induced depression (CUMS)	Mouse	20 mg/kg oral gavage	Depressive-like behavior ↓; synaptic proteins ↑	↑ Sirt1; ↓ NF-κB/NLRP3; ↑ Nrf2/HO-1	[72]
Corticosterone toxicity	Primary cortical cells	0.2–5 µM XN, 24 h pretreatment + 200 µM CORT × 96 h	Viability rescued; neuron/astrocyte balance	Nrf2-dependent; BDNF mRNA restored	[73]
Neuropathic pain (CCI)	Rat CCI	10–40 mg/kg XN, i.p., once daily × 10 days (starting day 1 post-CCI)	↑ thermal/mech thresholds	↓ TNF-α/IL-1β; ↓ p-ERK & NF-κB p65	[74]
Arthritis pain (CIA)	Mouse CIA	3-day i.p.	↓ flinches; ↑ thresholds; ↑ latency	↓ NLRP3; ↑ Nrf2; mito-ROS↓; AMPK binding; p-AMPK(Thr174) ↑	[75]
Ovariectomy-associated cognitive decline	OVX mice	30–60 mg/kg/day XN, oral gavage × 8 weeks	MWM/open field improved	miR-532-3p↓; Mpped1↑ (validated 3′UTR)	[76]
Iron overload injury	PC12; mouse	1–10 µM (PC12, 24 h pretreat); 10 mg/kg/day p.o. × 4 weeks (mouse)	Memory improved; ROS↓	AKT/GSK3β & Nrf2/NQO1 activation	[77]
Epilepsy (PTZ)	Mouse PTZ	20 mg/kg, −30 min	↓ onset/duration; ↓ mortality	↓ COX-2, TNF-α, NF-κB, TLR-4, IL-1β; ↓ MDA/NO; ↑ GSH/SOD; NTs normalized	[78]

MCAO, middle cerebral artery occlusion; ICH, intracerebral hemorrhage; KA, kainic acid; Aβ, amyloid-β; PP2A, protein phosphatase 2A; FXR, farnesoid X receptor; CUMS, chronic unpredictable mild stress; CCI, chronic constriction injury; CIA, collagen-induced arthritis; OVX, ovariectomized; PTZ, pentylenetetrazol; ROS, reactive oxygen species. Down arrow: decrease; Up arrow: increase.

**Table 6:** Molecular mechanisms underlying xanthohumol’s neuroprotection.

Pathway/Process	Mechanistic Modulations	Linked Functional Outcome	Behavioral Assays	References
Nrf2 antioxidant axis	↑ Nrf2/HO-1/NQO1/SOD; Nrf2 inhibitor blocks protection in cortical cells	Antioxidant defense; survival ↑	Depression tests; VA/ERG (retina)	[63,72,77]
NF-κB inflammatory signaling	↓ p-NF-κB/p65; cytokines ↓	Neuroinflammation ↓; behavior improved	Neuro scores; depression tests	[61,72]
NLRP3 inflammasome	NLRP3 markers ↓ (spinal cord/mPFC)	Analgesia; antidepressant-like effects	Pain batteries; CUMS	[72,75]
Autophagy/apoptosis (mTOR/LC3; Bax/Bcl-2)	Autophagy ↑; Bax/Bcl-2 shift	Cognitive rescue; Aβ↓	Morris water maze	[65]
Mitochondrial integrity	↑ Mfn-2 & Bcl-2; ↓ Apaf-1, caspase-3	Seizure protection	Seizure severity	[66]
Tau/kinase regulation	↓ tau pS404/396/262 via PP2A & GSK3β	AD pathology ↓	N/A	[64]
Synaptic glutamate release	Presynaptic GABA_A-dependent ↓ Ca^2+^ influx → CaM/PKA ↓ → glutamate release ↓	Anti-excitotoxic	N/A (synaptosomes)	[80]
AMPK/energy	AMPK binding & Thr174↑; Glut1/3↑; ATP, mitophagy↑	Analgesia; cognition ↑	Learning tasks; pain assays	[67,69,71,75]
Gut–brain axis	Microbiota composition shifts; proteome–microbiome correlation reversal at 0.5 mg/kg	Prevention-phase cognitive benefits	Learning/memory	[67,69]
miRNA regulation	miR-532-3p↓; Mpped1↑; validated 3′UTR	OVX cognition improved	Morris water maze; open field	[76]

Nrf2, nuclear factor erythroid 2-related factor 2; HO-1, heme oxygenase-1; NQO1, NAD(P)H quinone dehydrogenase 1; NF-κB, nuclear factor kappa-B; mPFC, medial prefrontal cortex; CUMS, chronic unpredictable mild stress; ERG, electroretinography; Mfn-2, mitofusin-2; AMPK, AMP-activated protein kinase; VA, visual acuity. Down arrow: decrease; Up arrow: increase.

## 5. Cardiovascular Effects of Xanthohumol

XN exhibits multiple cardioprotective actions through antioxidant, anti-inflammatory, metabolic, and endothelial-regulatory mechanisms. XN enhances Nrf2-dependent antioxidant defenses, reduces oxidative stress, and attenuates inflammatory signaling pathways such as NF-κB, contributing to protection against ischemia–reperfusion injury and endothelial dysfunction. It improves vascular tone by enhancing nitric oxide bioavailability, suppressing inducible nitric oxide synthase, and reducing reactive oxygen species, thereby supporting healthier endothelial function. An excellent review regarding XN’s cardiometabolic effects has been made available [83]. In this section, we focus on XN’s effects on endothelial functions.

### 5.1. Functional Cardiovascular Effects of XN

Anti-angiogenic effects: XN showed anti-angiogenic activity in various in vitro and in vivo models [24,84,85,86,87,88]. Using human HUVECs, XN reduced capillary-like structure formation on Matrigel and suppressed angiogenic signaling in vitro [24,84,85,89]. In vivo, reduced microvessel density and vascularization were observed in tumor xenograft and endometriosis models [24,86,87]. The magnitude of anti-angiogenic effects showed a dose-dependent pattern, with significant effects observed at concentrations as low as 5–10 micromolar [84,85,88].

Cellular viability and apoptosis: XN inhibited proliferations of endothelial cell and vascular smooth muscle cell [84,85,90,91]. XN could induce apoptosis with higher concentrations (10 micromolar) or prolonged exposure [84,85].

Migration and invasion inhibition: XN robustly inhibited migration and invasion of endothelial cells and vascular smooth muscle cells, as assessed by wound healing, Boyden chamber, and transwell assays [84,85,88,90,91]. Effects of XN against inflammation were observed both in the presence and absence of pro-inflammatory stimuli such as tumor necrosis factor alpha [24,88]. These inhibitory effects were dose- and time-dependent [84,85].

Endothelial barrier protection: XN was reported to attenuate tumor cell-mediated defects in the lymph endothelial barrier, reducing circular chemorepellent-induced defect (CCID) formation and suppressing adhesion molecule expression, showing a protective effect on endothelial integrity in the context of metastasis [92].

Inhibition of vascular calcification: In a rat model of vascular calcification induced by vitamin D3 and nicotine, XN reduced calcium deposition and alkaline phosphatase activity in calcified arteries. XN also decreased oxidative stress markers and improved arterial structure, while suppressing osteogenic transcription factors (BMP-2, Runx2) and preserving the vascular smooth muscle phenotype through upregulation of the Nrf2/Keap1/HO-1 antioxidant pathway [93].

Cardiac hypertrophy and fibrosis: Evidence indicates that XN attenuates pathological cardiac remodeling. In cardiac fibroblasts stimulated by TGF-β1, XN inhibits proliferation, differentiation, and collagen overproduction via modulation of the PTEN/Akt/mTOR signaling pathway, pointing to potential protection against fibrosis common in heart failure and hypertensive heart disease [2]. A consolidated summary of XN’s effects on endothelial angiogenesis, proliferation, migration, invasion, and barrier integrity across experimental models is presented in Table 7.

### 5.2. Molecular Mechanisms Underlying Cardiovascular Effects

Inhibition of NF-κB pathway: NF-κB inhibition is consistently observed across models of angiogenesis, migration and invasion, and endothelial barrier dysfunction [84,88,92]. This was demonstrated by reduced NF-κB activity, decreased expression of NF-κB target genes (such as adhesion molecules and cytokines), and suppression of downstream inflammatory and angiogenic responses [84,88,92].

Repression of Akt signaling: XN repressed Akt signaling, leading to decreased phosphorylation of Akt and its downstream targets, contributing to a reduction in cell survival, proliferation, and angiogenic capacity [84].

Activation of AMPK: XN was found to activate AMP-activated protein kinase in endothelial cells, mediated by calcium/calmodulin-dependent protein kinase kinase beta (CaMKKβ), resulting in decreased endothelial nitric oxide synthase (eNOS) phosphorylation and nitric oxide (NO) production [85]. These coordinated effects on metabolic and inflammatory signaling nodes are reflected in Table 8.

Other mechanisms: XN also downregulates epithelial–mesenchymal transition (EMT) markers, supporting its ability to inhibit migration, invasion, and barrier disruption [88,92]. Additional reported mechanisms include suppression of PI3K/Akt-associated signaling, cell cycle arrest, induction of apoptosis, suppression of VEGF, and downregulation of ICAM-1 across various cellular contexts [83,84,85,86,88,90,92].

Collectively, these findings highlight XN’s capacity to modulate multiple pro-pathogenic signaling networks central to vascular remodeling and cellular dysfunction. An integrated overview of these signaling pathways and their functional consequences is provided in Table 8.

## 6. Hepatic and Hepato-Renal Protections 

XN provides hepatoprotection across diverse liver injury models through activations of AMPK and Nrf2 and inhibition of NFκB, which coordinately reduce oxidative stress, suppress inflammation, regulate apoptosis, and prevent fibrosis.

### 6.1. Protection Across Liver Injury Models

In HFD-induced NAFLD, XN attenuated weight gain, improved glucose and insulin tolerance, and reduced hepatic lipid accumulation [94]. In CCl4-induced injury, XN significantly reduced liver weight, normalized plasma enzyme activities, and prevented histopathological alterations [95]. In acetaminophen-induced hepatotoxicity, XN could reduce mortality, ameliorate transaminase elevation, prevent glutathione depletion, and suppress lipid peroxidation [5,96]. XN demonstrated selective protection in the ischemia–reperfusion model. It showed inhibition of oxidative stress and almost completely blunted inflammatory responses [97]. XN showed significant reductions in aminotransferase levels, histological activity indices, and hepatic steatosis scores in an HCV-infected liver model. However, the reduction in HCV core protein expression was limited [98], suggesting that the protective effects were mediated primarily through anti-inflammatory and antioxidant mechanisms rather than direct antiviral activity. A comparative overview of liver injury models, XN dosing regimens, protective outcomes, and associated mechanisms is summarized in Table 9.

Several studies explicitly reported that XN’s hepatoprotection showed dose–response relationships. In vitro, XN showed dose-dependent inhibition of hepatic stellate cell activation between 0 and 20 μM, with no impairment of hepatocyte viability even at 50 μM [99,100]. This selective targeting of stellate cells versus hepatocytes suggests a favorable therapeutic window. In the aging model, effects were dose-dependent in most cases when comparing 1 versus 5 mg/kg/day [101]. The ethanol-induced liver damage model also demonstrated dose-dependent protection across multiple tissues [102].

### 6.2. Molecular Mechanisms of Hepatoprotection

Three major signaling pathways have been shown to be related to XN’s hepatoprotective mechanisms. The major molecular pathways underlying these hepatoprotective and metabolic effects are summarized in Table 10. AMPK activation emerged as a critical pathway, as shown, that increased AMPK phosphorylation (Thr72) and mRNA expression, which mediated the anti-steatotic effects [94]. Co-treatment with compound C, an AMPK inhibitor, completely abolished all protective effects mediated by XN. AMPK activation subsequently reduced expression of the lipogenic genes SREBP1c and ACC-1 [94].

Nrf2 activation represents another major mechanism. XN increased Nrf2 nuclear accumulation or transcription [5,94,96]. XN could covalently modify Keap1 [103], the negative regulator of Nrf2, thereby activating the Nrf2/xCT/GPX4 signaling pathway [98]. XN could also activate Nrf2 through the AMPK/Akt/GSK3β pathway [96], revealing crosstalk between AMPK and Nrf2 signaling.

NFκB inhibition was consistently observed across multiple studies. XN reduced NFκB nuclear accumulation [94], decreased hepatic NFκB activity [99], and blunted NFκB activation [97], as well as inhibiting NFκB and its dependent genes [99,100]. This inhibition resulted in reduced expression of NFκB-dependent pro-inflammatory genes, including TNF-α, IL-6, IL-1α, MCP-1, and ICAM-1 [94,99,100]. These coordinated anti-inflammatory effects across injury contexts are reflected in Table 10.

#### Other Molecular Targets

Oxidative stress reduction: XN consistently increased the reduced glutathione content while suppressing lipid peroxidation markers, including malondialdehyde [95,96,98], thiobarbituric acid reactive substances [95], and 4-hydroxynonenal conjugates. This was accompanied by preservation or enhancement of antioxidant enzyme activities, including superoxide dismutase [95,98], catalase [95,102], glutathione peroxidase [95,98], glutathione reductase [95], and glutathione S-transferase [95,97,102].

Cell-type-specific regulation of apoptosis: In hepatocytes, XN reduced apoptotic nuclei and suppressed pro-apoptotic pathways, including JNK phosphorylation and mitochondrial translocation, Bax translocation, cytochrome c and AIF release, and caspase-3 activation [96,98,101]. Conversely, in activated hepatic stellate cells, XN promoted apoptosis [99,100], contributing to anti-fibrotic effects. This differential regulation—protecting hepatocytes while eliminating activated stellate cells—represents a therapeutically favorable mechanism.

Fibrosis inhibition: XN reduced expression of transforming growth factor-β1 [98], a master regulator of fibrogenesis, along with downstream targets collagen type I and α-smooth muscle actin [99]. Direct inhibition of hepatic stellate cell activation , combined with induction of apoptosis in already-activated stellate cells [99,100], provides complementary anti-fibrotic mechanisms.

### 6.3. Integrated Hepato-Renal Protection

XN’s protective effects extend beyond the liver to the broader hepato-renal axis, reflecting the shared vulnerability of hepatic and renal tissues to oxidative stress, inflammation, and metabolic imbalance. In ethanol-induced oxidative injury models, XN provided dose-dependent protection not only in the liver but also in the kidney, lung, heart, and brain, reducing lipid peroxidation, preserving antioxidant enzyme activity, and normalizing injury biomarkers [102]. These findings indicate that XN’s cytoprotective actions are systemic rather than organ-restricted.

Mechanistically, the same pathways mediating renal protection are involved in hepatoprotection. Antioxidant capacity and metabolic resilience are enhanced by AMPK and Nrf2 activation, while suppression of cytokine-driven tissue injury is mediated by inhibition of NF-κB signaling. Oxidative stress, mitochondrial dysfunction, and inflammation are central drivers of renal damage; thus, modulation of these pathways across organs supports XN’s classification as a broad-spectrum cytoprotective agent.

**Table 9:** Hepatoprotection of xanthohumol.

Injury/Disease Model (System)	Dose/Regiment	Key Protective Outcomes	Mechanisms	Ref.
HFD-induced NAFLD (rat)	Oral 20–30 mg/kg (per study)	↓ body-weight gain; ↓ fat pads; ↓ fasting glucose/insulin; ↓ hepatic TGs/TC/FFAs; ↓ ALT/AST; ↓ lipid droplets	AMPK required: compound C abolished protection	[94]
CCl_4_ acute injury (rat)	Single CCl_4_ ± XN	↓ liver weight; normalized LDH/GOT/GPT (*p* < 0.05); ↓ histopathology; ↑ GSH; ↓ TBARS/H_2_O_2_	↑ SOD, catalase, GPx, GR, GST	[95]
APAP (acetaminophen) toxicity (mouse and hepatocyte models)	XN pretreatment	↓ mortality; ↓ ALT/AST; prevented GSH depletion; ↓ MDA; improved histology	Nrf2 via AMPK/Akt/GSK3β	[96]
CCl_4_ injury/inflammation–fibrosis (mouse)	XN co-treatment	Blunted pro-inflammatory & profibrogenic genes; ↓ NF-κB activity	↓ serum transaminases; ↓ necrosis	[99]
APAP-induced injury with ferroptosis focus (HepaRG cells and mouse)	XN pretreatment	Ameliorated AILI in vitro & in vivo	Nrf2/xCT/GPX4 via Keap1 cysteine modification	[98]
Hepatitis C-associated liver injury (*Tupaia belangeri*)	Systemic XN	↓ aminotransferases; ↓ histological activity index; ↓ steatosis; ↓ TGF-β1	HCV core ↓ not significant (hepatoprotection > direct antiviral)	[48]
Warm ischemia–reperfusion (mouse)	XN pre-I/R	↓ oxidative stress; ↓AKT & NF-κB activation; ↓ IL-1α/IL-6/MCP-1/ICAM-1	No significant change in acute necrosis (H&E/TUNEL/ALT-AST)	[97]
NASH/hepatic fibrosis (mouse; hepatic stellate cell culture)	0–20 µM (HSC), ≤50 µM (hepatocytes)	↓ HSC activation; ↑ apoptosis in activated HSC; ↓ hepatic inflammation & profibrotic genes	Hepatocytes viable up to 50 µM	[100]
Aging-related alterations (aging rat)	1 vs. 5 mg/kg/day	Dose-dependent modulation of apoptosis/oxidative stress/inflammation (*p* < 0.05)	AIF, BAD, BAX, Bcl-2, eNOS, HO-1, IL-1β, NF-κB2, PCNA, SIRT1, TNF-α	[101]
Ethanol-induced oxidative damage (rat; multi-organ assessment)	Dose per study	Dose-dependent protection (liver, kidney, lung, heart, brain)	Enzymes (SOD, catalase, GST); GOT/GPT/LDH; lipid peroxidation	[102]
Clinical safety/PK (human, Phase I XMaS trial)	Oral XN	Tolerated in healthy adults; safety/tolerability profiled	Formulation likely important	[5]
Metabolic comorbidity context (Review)	N.A.	Summarizes benefits in hyperlipidemia, obesity, T2DM	Mechanistic overlap with hepatic AMPK/Nrf2	[104]

NAFLD, non-alcoholic fatty liver disease; NASH, non-alcoholic steatohepatitis; HFD, high-fat diet; APAP, acetaminophen; HSC, hepatic stellate cell; ALT, alanine aminotransferase; AST, aspartate aminotransferase; TGs, triglycerides; TC, total cholesterol; FFAs, free fatty acids; GSH, glutathione; TBARS, thiobarbituric acid-reactive substances; N.A., not available. Down arrow: decrease; Up arrow: increase.

**Table 10:** Mechanistic actions of xanthohumol on hepatic and renal protective effects.

Pathway Readouts	Downstream Effects	Functional Outcome	Refs.
AMPK activation; compound C abrogates effects	↓ SREBP-1c, ↓ ACC-1; improved metabolic parameters	Anti-steatosis; cytoprotection	[94,96]
Nrf2 activation;	↑ GSH/SOD/CAT; xCT/GPX4engaged	Antioxidant defense; anti-ferroptosis	[94,96,98]
Keap1→Nrf2; Covalent modification of Keap1 cysteines	Stabilizes Nrf2 → xCT/GPX4	Blocks hepatic ferroptosis in APAP	[98]
NF-κB inhibition	↓ TNF-α, IL-6, IL-1α; ↓ MCP-1, ICAM-1	Anti-inflammatory; limits I/R cytokines	[94,97,99,100]
↑AMPK→Akt→GSK3β→	Nrf2 activation; cytoprotection	Integrated stress response	[96]
↓AKT (I/R context)	↓ inflammatory gene induction	Selective anti-inflammatory benefit	[97]
↓ ROS/LPO ↑ GSH; ↓ MDA/TBARS/H_2_O_2_; ↓ 4-HNE	Preserved SOD, CAT, GPx, GR, GST; ↑ HMOX1	Limits hepatocyte injury	[48,94,95,96,97,98,99,100,101,102]
Hepatocytes: ↓ JNK; ↓ Bax translocation; ↓ Cyt-c/AIF; ↓ Casp-3.	Cell-type specific apoptosis regulation: hepatocyte apoptosis ↓; HSC apoptosis ↑	Anti-injury + anti-fibrotic	[48,96,100,101]
Fibrosis signaling; ↓ TGF-β1; ↓ collagen I; ↓ α-SMA; ↓ HSC activation	↓ Fibrogenesis, Matrix remodeling reduced	Anti-fibrotic	[48,99,100]
↓ SREBP-1c/ACC-1; ↓ TGs/TC/FFAs	Lipogenesis inhibition;↓ hepatic lipid droplets	Anti-steatosis	[94]
Clinical translation/safety; Phase I safety; metabolic disease rationale	Supports human feasibility	Guides formulation & dosing work	[5,104]

AMPK, AMP-activated protein kinase; ACC-1, acetyl-CoA carboxylase-1; SREBP-1c, sterol regulatory element-binding protein-1c; Nrf2, nuclear factor erythroid 2-related factor 2; Keap1, Kelch-like ECH-associated protein 1; NF-κB, nuclear factor kappa-B; HSC, hepatic stellate cell; APAP, acetaminophen; I/R, ischemia–reperfusion; ROS, reactive oxygen species; MDA, malondialdehyde; TBARS, thiobarbituric acid-reactive substances; HMOX1, heme oxygenase-1. Down arrow: decrease; Up arrow: increase.

## 7. Metabolic Modulation 

XN is a multifunctionally metabolic modulator, coordinating gut–liver signaling, cellular energy sensing, and lipid–glucose homeostasis rather than acting on a single pathway. In obesity and NAFLD models, XN improves whole-body insulin sensitivity by activating AMPK and SIRT1, which enhances GLUT4 translocation, suppresses hepatic gluconeogenesis by decreasing PEPCK and G6Pase, and shifts mitochondrial metabolism toward oxidation [105,106]. These upstream changes translate into lower fasting glucose, improved insulin action, and reduced hepatic triglyceride accumulation, accompanied by decreased de novo lipogenesis-related genes such as *Srebp-1c*, *Fas*, and *Acc*) and increased β-oxidation-related genes *Cpt-1* and *Pparα* [105,106].

XN also directly remodels adipose tissue. It inhibits adipogenesis-related genes such as *Pparγ* and *C/ebpα* and promotes browning/thermogenesis-related gene *Ucp1*, linking AMPK/SIRT1 activation to smaller, more oxidative fat depots [98,99]. In vivo, these effects are associated with higher energy expenditure, resistance to HFD-induced weight gain, and enhanced mitochondrial biogenesis (PGC-1α/SIRT1) and BAT thermogenesis [105,106]. Suppression of NF-κB and cytokines (TNF-α, IL-6) and activation of Nrf2 antioxidant signaling further relieve insulin resistance and restore hepatic redox balance [105,106].

A defining feature of XN’s metabolic efficacy is its reliance on an intact gut microbiome. XN improved insulin resistance in conventionally colonized mice but not in germ-free animals, implicating microbial transformation and microbiota remodeling as essential mediators [107]. This gut–liver axis underlies simultaneous reductions in inflammation, lipogenesis, and dysglycemia as hepatic NF-κB signaling quiets and AMPK/Nrf2 pathways dominate [105,107].

Translationally, XN shows promise but is limited by low oral bioavailability due to hydrophobicity and first-pass metabolism. Early human studies confirm safety and indicate improvements in CRP, glycemia, and microbiome composition [5,105]. Advances in formulation (nanocarriers, lipid systems, inclusion complexes) will be crucial for stabilizing parent XN and enabling rigorous clinical testing in NAFLD/NASH, type 2 diabetes, obesity, and metabolic syndrome [105,108].

In summary, XN drives a microbiota-dependent, AMPK/SIRT1-centered realignment of lipid and glucose metabolism while suppressing inflammatory stress, an integrated mechanism that supports advancing optimized formulations into larger outcome-focused human trials [105,106,107]. A structured overview of XN’s metabolic targets, underlying mechanisms, representative outcomes, and dose ranges across preclinical and human studies is provided in Table 11.

## 8. Dermatological and Joint-Protective Effects

Accumulating evidence supports XN as a cytoprotective compound capable of attenuating oxidative stress, suppressing inflammatory signaling, preserving extracellular matrix (ECM) integrity, and limiting tissue degeneration in both cutaneous and joint-related contexts. These properties are particularly relevant in pathological conditions driven by chronic inflammation, oxidative injury, and dysregulated matrix remodeling.

### 8.1. Protection of Dermatological and Joint Conditions

Photodamage and Photoaging: XN exhibits protective activity in experimental systems relevant to photodamage and photoaging, particularly through preservation of ECM structure. In dermal fibroblast models, XN directly inhibited elastase and matrix metalloproteinases (MMPs) while stimulating biosynthesis of fibrillar collagens, elastin, and fibrillins, indicating a pro-matrix profile under inflammatory stress conditions [109]. Complementary material-based models further demonstrate that XN can influence collagen integrity under ultraviolet (UV) irradiation, supporting a role in mitigating UV-associated matrix degradation [110]. Together, these findings suggest that XN interferes with key enzymatic and oxidative processes that contribute to progressive dermal structural decline.

Anti-Acne and Inflammatory Skin Responses: XN and hop-derived extracts show activities against biological drivers of acne and inflammatory skin responses. In vitro studies of hop components demonstrated antibacterial, anticollagenase, and antioxidant activity relevant to acne-associated inflammation and connective tissue damage [8]. Consistent with these findings, hop extract exhibited antimicrobial effects against Propionibacterium acnes and multiple Staphylococcus aureus strains (e.g., S. aureus ATCC 29213, S. aureus ATCC 25923, S. aureus 2407, and an MRSA clinical isolate), alongside antioxidant activity in skin-relevant models [111]. At the mechanistic level, XN suppresses inflammatory mediator production in activated macrophages, providing a plausible molecular basis for its anti-inflammatory effects in cutaneous contexts [112]. While clinical efficacy data remain limited, these results support further investigation of XN in inflammation-driven skin disorders.

Extracellular Matrix Preservation and Tissue Integrity: Beyond photoaging, XN exerts matrix-preserving effects that support long-term tissue integrity. In vitro studies demonstrate that XN suppresses collagen-degrading enzymes while promoting synthesis of structural ECM components in dermal fibroblasts, including fibrillar collagens and elastin-associated proteins [109]. These actions align with broader anti-inflammatory effects of XN on signaling pathways relevant to connective tissue homeostasis and joint inflammation, suggesting shared protective mechanisms across skin and musculoskeletal systems [112].

Barrier Function: XN’s antioxidant and anti-inflammatory properties may further contribute to epidermal resilience and modulation of stress-associated skin responses. In a randomized, blinded half-side comparison study, use of a botanical cleansing lotion was associated with changes in facial sebum levels and erythema, indicating potential benefits for skin homeostasis under mild inflammatory conditions [113] .

Arthritis and Joint Inflammation: In joint disease models, XN has demonstrated anti-inflammatory and tissue-protective activity across multiple experimental paradigms, including surgically induced osteoarthritis, obesity-associated osteoarthritis, and systemic inflammatory arthritis [114,115,116]. In adjuvant-induced arthritis, micellar-solubilized XN reduced paw swelling more effectively than native XN and achieved anti-inflammatory efficacy comparable to diclofenac [115]. In osteoarthritis-focused studies, XN attenuated IL-1β- and palmitate-induced inflammatory responses in chondrocytes, suppressed catabolic enzymes contributing to cartilage degradation, and improved cartilage integrity in vivo [114,116].

### 8.2. Molecular Mechanisms Underlying Cutaneous and Joint-Protective Effects

Across arthritis models, XN consistently suppressed inflammatory signaling at the molecular level. Chen et al. reported broad inhibition of pro-inflammatory mediators—including iNOS, nitric oxide, TNF-α, IL-6, and COX-2—alongside reduced MMP-13 expression and restoration of cartilage matrix components such as type II collagen and aggrecan [114]. Khayyal et al. similarly observed decreases in systemic inflammatory markers (TNF-α, IL-6, and CRP) and oxidative stress indicators (myeloperoxidase activity and lipid peroxidation) [115]. Sun et al. extended these findings by showing suppression of cytokines and ECM-degrading enzymes in palmitate-treated chondrocytes, together with improved mitochondrial function [116].

Mechanistic analyses converged on NF-κB inhibition as a central pathway, though upstream regulatory mechanisms differed between studies. Chen et al. identified coordinated activation of the Nrf2 antioxidant pathway and inhibition of NF-κB signaling in inflamed chondrocytes [114]. Sun et al. instead described an AMPK-dependent mechanism in which XN improved mitochondrial biogenesis, reduced mitochondrial dysfunction, blocked NLRP3 inflammasome activation, and thereby indirectly suppressed NF-κB signaling [116]. These findings indicate that XN engages distinct upstream nodes—Nrf2 versus AMPK—depending on whether the pathological driver is cytokine-mediated inflammation or metabolic stress, while converging on the same downstream anti-inflammatory targets.

### 8.3. Formulation and Delivery to Skin

Native XN is hydrophobic and sensitive to light and oxidation, properties that may limit topical efficacy if not addressed through formulation strategies. Nano- and carrier-based delivery systems improve cutaneous penetration, enhance stability, and enable lower effective doses with improved local exposure [117]. Micellar-solubilized XN demonstrated significantly greater anti-inflammatory efficacy than native XN in vivo, suggesting that optimized formulations may be required for therapeutic translation and may explain variability across preclinical outcomes [115]. With optimized delivery systems, XN holds strong translational potential for photoaging prevention, acne management, and arthritis treatment—areas where its multi-target actions could offer meaningful therapeutic advantages.

Together, the evidence supports XN as a modulator of joint inflammation and cartilage degeneration across both osteoarthritis and inflammatory arthritis models [114,115,116]. However, translational gaps remain. Dosing and treatment duration were inconsistently reported, quantitative effect sizes were variably described, adverse effects were not systematically assessed, and no human efficacy data are available. Nonetheless, the convergent anti-inflammatory, antioxidant, and matrix-preserving actions across diverse arthritis paradigms position XN as a promising candidate for further translational research and eventual clinical exploration.

## 9. XN Studies on Human Subjects

To date, completed and ongoing human studies investigating hop-derived XN, XN-enriched extracts, or hop-containing formulations collectively suggest that XN is pharmacokinetically tractable, short-term safe, and biologically active in humans, but that evidence for consistent clinical benefit remains preliminary.

Initial pharmacokinetic investigations in healthy adults demonstrated that XN enters the circulation after oral intake, with maximal plasma levels detected within several hours and elimination occurring over an extended time frame on the order of one day [6]. Circulating XN was detected predominantly in metabolized, conjugated forms rather than as free aglycone, and no major safety concerns were identified across a broad range of single-dose exposures. Comparable pharmacokinetic behavior was observed in menopausal women receiving a standardized hop extract containing XN and related prenylated flavonoids, where absorption was gradual, apparent half-lives were prolonged, and no adverse effects on endocrine or coagulation markers were reported [7]. Together, these findings establish a favorable pharmacokinetic and short-term safety profile for XN across a wide range of single-dose exposures.

The strongest evidence for repeated-dose safety comes from the XN Microbiome and Signature (XMaS) Phase I trial, in which healthy adults received 24 mg/day of highly purified XN (99.8%) for eight weeks [1,5]. In this triple-masked, placebo-controlled study, XN was well tolerated, with no dose-limiting toxicities, serious adverse events, or clinically meaningful changes in laboratory values, vital signs, body weight, or health-related quality-of-life measures. These data support the short-term safety of XN at nutraceutical-like doses and provide an essential foundation for disease-focused Phase II studies. Consistent with this safety profile, a pharmacokinetic interaction study of a hop botanical supplement containing XN in peri- and postmenopausal women did not identify clinically relevant effects on the metabolism of probe drugs, further supporting tolerability in populations likely to use dietary supplements [118].

Beyond safety and pharmacokinetics, several controlled human interventions indicate that XN exerts measurable biological effects at both moderate and very low doses. In a controlled crossover study using an XN-containing beverage, daily intake in the range of 6–24 mg reduced biomarkers of oxidative DNA damage and DNA strand breaks induced by dietary carcinogens, consistent with chemoprotective mechanisms observed in preclinical models [11]. Notably, immune-modulating effects have also been observed at microgram-level exposures. Acute crossover studies in healthy adults demonstrated that beverages or extracts providing approximately 0.125 mg XN attenuated ex vivo cytokine production (IL-1β, IL-6, and TNF-α) and dampened Toll-like receptor 4 signaling in peripheral blood mononuclear cells following bacterial stimulation [3,4]. While these studies do not demonstrate clinical efficacy, they provide consistent evidence that XN can interact with inflammatory and innate immune pathways in humans at doses achievable through diet or supplementation.

Disease-focused clinical investigations are now emerging. A Phase II randomized trial in adults with Crohn’s disease is underway to evaluate the safety, biological activity, and disease-related signatures of XN at a dose of 24 mg/day, building directly on the XMaS Phase I safety data [12]. Additional ongoing or recently completed trials are exploring formulation-dependent bioavailability, metabolic effects, and immune responses following XN intake, although results from several of these studies remain unpublished [9,10]. The most striking clinical signal reported to date comes from a randomized trial in critically ill COVID-19 patients, in which short-term administration of a high-dose XN-rich *Humulus lupulus* extract as an adjunct to standard care was associated with lower 28-day mortality, shorter ICU stays, and greater reductions in inflammatory and coagulation markers compared with standard care alone [119]. While intriguing, this finding arose in a single, relatively small trial conducted in a highly specific, high-risk clinical context and requires independent replication. An overview of completed and ongoing human studies of XN, including pharmacokinetics, safety, formulation, biological endpoints, and clinical trial status, is summarized in Table 12.

Despite these encouraging findings, the current clinical evidence base for XN has several limitations. Most studies are small (typically enrolling 20–50 participants), single-center, and early-phase, and are designed primarily to assess pharmacokinetics, safety, or mechanistic biomarkers rather than robust clinical endpoints such as long-term disease activity, hospitalization, or mortality. Many immune studies rely on acute crossover designs and ex vivo stimulation of isolated blood cells, which provide valuable mechanistic insight but do not establish whether XN meaningfully improves clinical outcomes in vivo. In addition, there is substantial heterogeneity in dose, formulation, duration, and route of administration across studies, ranging from microgram-level beverages to repeated oral dosing in the tens of milligrams per day and multi-milligrams per kilogram dosing in ICU settings. Follow-up periods are generally short, and long-term safety data in diverse populations, including older adults, individuals with comorbidities, and those on polypharmacy, remain limited.

Importantly, these human studies were not designed to assess disease-specific efficacy and relied primarily on pharmacokinetic, safety, or short-term biomarker endpoints. Sample sizes were modest, intervention durations were limited, and outcomes were not powered to detect clinical benefit. Accordingly, while these trials support the biological activity and tolerability of XN in humans, they do not provide evidence for therapeutic efficacy in any disease context.

## 10. Limitations and Future Directions

Despite the substantial body of preclinical evidence supporting its pleiotropic biological effects, there are several important limitations that constrain XN’s current translational interpretation. A major limitation of the literature summarized in this review is the heavy reliance on in vitro systems and animal models. While these studies consistently demonstrate modulation of conserved signaling pathways, including the NF-κB, PI3K/AKT/mTOR, Nrf2, AMPK, and MAPK cascades, the extent to which these mechanisms translate to human physiology remains incompletely defined. Experimental heterogeneity across studies, including differences in disease models, dosing strategies, exposure durations, and outcome measures, further complicates direct comparison and limits the establishment of standardized therapeutic windows. Moreover, many experimental studies employ concentrations of XN that may not be readily achievable in humans following oral administration of the native compound.

Another key limitation relates to pharmacokinetic constraints. Native XN is hydrophobic and undergoes rapid metabolism, resulting in limited systemic bioavailability. Although formulation strategies such as micellar delivery systems have demonstrated improved absorption and circulating plasma levels, clinical evidence remains largely restricted to short-term safety, tolerability, and biomarker-based outcomes. Long-term safety, tissue distribution, metabolite-specific activity, and potential interactions with pharmaceuticals or other nutraceuticals remain insufficiently characterized. In addition, potential sources of interindividual variability—including sex-specific responses, microbiome composition, and disease-stage-dependent effects—have not been systematically addressed across studies.

In addition, there are several technical hurdles in the extraction and purification of XN from hop cones. The standard workflow includes organic solvent extraction using methanol or ethanol, followed by HPLC-based purification, and LC-MS/MS characterization for structural confirmation. However, XN is highly thermally and light-sensitive, making it prone to isomerization to form less bioactive byproducts such as isoxanthohumol [120,121]. This degradability necessitates specialized strategies to enhance its long-term storage. To date, there is a lack of universally available reference standards and the frequent formation of artifacts during processing. This significantly imposes analytical challenges for HPLC-UV in distinguishing XN from minor related prenylflavonoids and plant matrix interference. The poor aqueous solubility of XN further complicates the extraction and limits the overall yield. It is therefore pivotal to optimize parameters such as temperature, pH, oxygen concentration, and the presence of metallic ions to minimize degradation and enhance extraction efficiency [120].

The current human subject studies consistently support its short-term safety and demonstrate reproducible interactions with inflammatory and oxidative stress pathways, but robust, multicenter randomized trials demonstrating consistent clinical benefits are still lacking. Future research on XN should prioritize the transition from small, mechanistic and pharmacokinetic studies toward larger, multicenter randomized clinical trials with clearly defined doses, standardized formulations, and clinically meaningful endpoints in specific patient populations, such as inflammatory bowel disease, metabolic syndrome, viral infections, or critical illness. Systematic dose–response and formulation studies (e.g., native versus micellar XN, pure XN versus XN-rich hop extracts, and oral versus parenteral delivery) will be essential to define a realistic therapeutic window and to disentangle the effects of XN from those of other hop-derived constituents. In this regard, several ongoing clinical trials are expected to provide important insight into XN bioavailability, metabolic effects, oxidative stress modulation, microbiome interactions, and safety in human populations [9,10,11,12,13]. Although results from these studies are not yet available, they represent a necessary step toward addressing current translational gaps and informing rational study design for future intervention trials.

Parallel long-term safety studies and formal drug interaction trials will also be necessary to support broader clinical use. Mechanistic integration of microbiome profiling, metabolomics, and immunophenotyping may further help identify responder subgroups and clarify how interindividual differences in prenylflavonoid metabolism influence efficacy. Accumulating preclinical and early clinical evidence suggests that XN may modulate gut microbiota composition and downstream gut–host signaling pathways, thereby influencing metabolic, inflammatory, and neurocognitive outcomes [5,12,67,68,69,107]. These observations highlight the potential importance of microbiome-dependent mechanisms in mediating interindividual variability in response to XN. Accordingly, future studies integrating microbiome profiling, metabolomics, and other multi-omics approaches may provide valuable insight into responder phenotypes and support the development of precision nutrition strategies. Finally, evaluation of XN as an adjunct to established therapeutic regimens—including anti-inflammatory, antiviral, and anticancer agents—represents a promising avenue to leverage its multi-target mechanisms while potentially mitigating toxicity and resistance.

## 11. Conclusions

Collectively, the available evidence positions XN as a pleiotropic bioactive compound with therapeutic relevance across oncologic, metabolic, inflammatory, infectious, neurodegenerative, hepatic, renal, dermatological, and musculoskeletal disorders. Rather than acting through a single target, XN consistently engages a conserved network of cellular stress-response pathways—most notably AMPK activation, Nrf2-mediated antioxidant signaling, and NF-κB inhibition. Through these interconnected mechanisms, XN coordinately regulates oxidative stress, mitochondrial integrity, inflammatory cascades, apoptosis, ferroptosis, and extracellular matrix remodeling.

Across diverse disease models, this mechanistic convergence yields reproducible functional outcomes: suppression of pathological inflammation, preservation of tissue structure, and selective modulation of cell survival. In cancer, XN downregulates proliferative and metastatic molecules while inducing apoptosis and inhibiting angiogenesis. In non-oncologic contexts—including the liver, kidney, neural tissue, skin, and joints—XN provides cytoprotection by attenuating oxidative injury, dampening inflammatory signaling, and maintaining metabolic and mitochondrial homeostasis. Importantly, XN often demonstrates beneficial cell-type selectivity, supporting survival of parenchymal cells while suppressing activated, pathogenic, or profibrogenic cell populations. AMPK–Nrf2 activation and NF-κB suppression provide a unifying explanation of its protection against toxic, ischemic, inflammatory, and metabolic insults, reinforcing the concept of XN as a systems-level modulator of cellular stress rather than a tissue-restricted agent.

Despite this compelling preclinical profile, translational gaps between experimental models and clinical application remain, as human evidence is currently limited largely to safety and tolerability studies. Many investigations lack standardized dosing, comprehensive pharmacokinetic characterization, and long-term clinical outcome data. Native XN also exhibits poor oral bioavailability, although emerging nano- and micellar formulations show promise in enhancing systemic exposure and efficacy. Future efforts should prioritize optimized delivery strategies, rigorous pharmacokinetic and mechanistic evaluation, and well-designed clinical trials to establish therapeutic windows and disease-specific benefits.

With continued refinement of formulations and clinical testing, XN holds strong potential as a clinically meaningful adjunct or nutraceutical for chronic inflammatory, metabolic, and degenerative diseases.

## Figures and Tables

**Table 7:** Xanthohumol effects on endothelial function.

Cellular Function	Concentration Range (µM)	Biological Effect	Molecular Mechanism	Refs.
Angiogenesis (tube formation, microvessel density)	2.5–40 (in vitro); 100 (in vivo)	Significant inhibition (often dose-dependent); for example, microvessel density decreased, tube formation decreased	AMPK activation; NF-κB/Akt (PI3K-dependent) signaling suppression	[84,85,88]
Proliferation/viability	5–20	Viability decreased, proliferation decreased (statistically significant in several studies)	AMPK activation, cell cycle arrest, apoptosis induction	[84,85]
Migration/invasion	5–20	Migration decreased, invasion decreased (statistically significant, dose-dependent)	NF-κB, VEGF, Akt, EMT marker suppression	[84,85,88]
Barrier function	Low	CCID formation decreased, adhesion decreased	NF-κB, ICAM-1, EMT marker suppression	[88,92]

AMPK, AMP-activated protein kinase; NF-κB, nuclear factor kappa-B; PI3K, phosphoinositide 3-kinase; Akt, protein kinase B; VEGF, vascular endothelial growth factor; EMT, epithelial–mesenchymal transition; ICAM-1, intercellular adhesion molecule-1; CCID, circular chemorepellent-induced defect.

**Table 8:** Mechanistic action of cardiovascular protection by xanthohumol.

Signaling Pathway	Target Proteins	Downstream Effects	Functional Outcome	Refs.
NF-κB	Decreased NF-κB activity and cytokine expression	Decreased NF-κB activity, decreased adhesion molecule/cytokine expression	Decreased migration, invasion, angiogenesis, inflammation	[24,84,88,92]
Akt	Akt, downstream survival/proliferation proteins	Decreased Akt phosphorylation/activity	Decreased proliferation, survival, angiogenesis	[84,85]
AMPK	AMPK, eNOS	Increased AMPK phosphorylation/activity, decreased eNOS phosphorylation	Decreased angiogenesis proliferation	[85]
PI3K	PI3K	Reduced PI3K/Akt-associated signaling	Decreased lesion growth, vascularization	[87]
EMT/Adhesion	Decreased ICAM-1, SELE, paxillin, MCL2, S100A4	Suppressed expression/activity	Decreased adhesion, migration, EMT, metastasis	[88,92]

Footnote: NF-κB, nuclear factor kappa-B; Akt, protein kinase B; AMPK, AMP-activated protein kinase; eNOS, endothelial nitric oxide synthase; PI3K, phosphoinositide 3-kinase; EMT, epithelial–mesenchymal transition; SELE, selectin E; MCL2, myosin light chain 2; ICAM-1, intercellular adhesion molecule-1.

**Table 11:** Metabolic modulation of xanthohumol.

Focus Area	Core Mechanisms	Outcomes	Dose/Model	Refs.
Glucose uptake & insulin sensitivity	AMPK ↑ with SIRT1 support → GLUT4 translocation ↑; hepatic gluconeogenesis ↓ (PEPCK, G6Pase)	Fasting glucose ↓; insulin sensitivity ↑	5–60 mg/kg/day (HFD/db/db rodents); 5–20 µM (cells)	[105,106]
Lipid metabolism/NAFLD	Lipogenesis ↓ (SREBP-1c/FAS/ACC); β-oxidation ↑ (CPT-1/PPARα)	Hepatic TGs ↓; steatosis↓; LDL/TGs ↓	10–60 mg/kg/day (rodents); 5–20 µM (hepatocytes)	[105,106]
Adipose remodeling	Adipogenesis ↓ (PPARγ/C/EBPα); browning/thermogenesis ↑ (UCP1; PGC-1α/SIRT1)	Fat mass ↓; more oxidative depots	Cell (3T3-L1); HFD mice (oral XN per study)	[106]
Energy expenditure & mitochondria	Mitochondrial biogenesis ↑ (AMPK/PGC-1α); BAT thermogenesis ↑	Energy expenditure ↑; resistance to HFD weight gain	Rodent HFD models; formulation studies	[104,105]
Anti-inflammatory & antioxidant tone	NF-κB/TNF-α/IL-6 ↓; Nrf2/ARE ↑	Meta-inflammation ↓; redox balance stabilized	As above	[105,106]
Human exposure & safety	Phase I exposure/safety; early biomarker shifts	Safe ≤ 180 mg/day; oxidative-stress markers ↓; CRP trend ↓	12–180 mg/day, 3–8 weeks	[5,6]
Formulation & delivery	Nano/lipid carriers, inclusion complexes	Bioavailability ↑; steadier parent XN exposure	N.A.	[108]

AMPK, AMP-activated protein kinase; SIRT1, sirtuin 1; GLUT4, glucose transporter type 4; PEPCK, phosphoenolpyruvate carboxykinase; G6Pase, glucose-6-phosphatase; NAFLD, non-alcoholic fatty liver disease; CPT-1, carnitine palmitoyltransferase-1; BAT, brown adipose tissue; ARE, antioxidant response element. Down arrow: decrease; Up arrow: increase.

**Table 12:** Biological effects of xanthohumol-enriched hop extracts or hop-derived preparations in skin-related models.

Study Description	Cohort (*n*)	XN Source & Dose	Study Design & Duration	Key Findings	Ref.
Human pharmacokinetics of xanthohumol	Healthy adults (*n* = 48)	Pure XN, 20–180 mg single oral dose	Single-dose pharmacokinetic study; serial sampling up to 120 h	Oral absorption with biphasic profile; half-life: ~18–20 h; circulating forms mainly conjugated XN and isoxanthohumol; no major safety signals	[6]
Pharmacokinetics of prenylated hop phenols	Menopausal women (*n* = 5)	Standardized hop extract containing XN and other prenylflavonoids	Dose-escalation pharmacokinetic and safety study	Slow absorption and long half-lives (>20 h); no effects on sex hormones or coagulation parameters	[7]
Plasma appearance of xanthohumol	Healthy adults (*n* = 12)	86 or 172 mg native vs. micellar XN	Randomized four-way crossover; single dose	Study completed; designed to compare exposure (AUC, Cmax) of native versus micellar XN; results pending publication	[9]
Effects of xanthohumol on resting energy expenditure	Healthy young women (*n* = 16)	172 mg micellar XN	Triple-blind crossover; acute dosing	Completed in 2024; assessed resting energy expenditure and substrate oxidation; results pending	[10]
Xanthohumol Microbiome and Signature (XMaS), Phase I	Healthy adults (*n* = 30)	24 mg/day pure XN (99.8%)	Triple-masked, randomized, placebo-controlled; 8 weeks	Well tolerated; no serious adverse events or clinically meaningful changes in laboratory parameters, vital signs, or body weight	[12]
Prevention of oxidative DNA damage by XN	Healthy adults (*n* = 64)	XN beverage providing 6–24 mg/day	Quadruple-blind crossover	Reduced markers of oxidative DNA damage and DNA strand breaks	[11]
Low-dose XN and PBMC inflammatory response	Healthy adults (*n* = 14)	Beverage containing 0.125 mg XN	Single-blind crossover; acute intake	Reduced ex vivo IL-1β, IL-6, and TNF-α release after PBMC stimulation	[4]
Xanthohumol-rich hop extract and TLR4 signaling	Healthy women (*n* = 12)	XN-rich hop extract (~0.125 mg XN)	Randomized crossover; acute intake	Attenuated LPS-induced TLR4 signaling and cytokine production in PBMCs	[3]
Xanthohumol Microbiome and Signature (XMaS), Phase II (Crohn’s disease)	Adults with Crohn’s disease	24 mg/day pure XN	Phase II, triple-masked RCT; 8 weeks	Ongoing; evaluating disease activity indices, inflammatory markers, microbiome, and bile acid profiles	[12]
Hop botanical dietary supplement metabolism and safety	Peri- and postmenopausal women (*n* = 16)	Standardized hop extract containing XN	Phase I pharmacokinetic interaction study	No clinically relevant effects on CYP-mediated drug metabolism observed	[118]

XN, xanthohumol; AUC, area under the concentration–time curve; Cmax, maximum plasma concentration; PBMC, peripheral blood mononuclear cell; TLR4, Toll-like receptor 4.

## Data Availability

The original data presented in this review paper are searched from and openly available in multiple scientific databases, including PubMed, Google Scholar, ScienceDirect, Wiley Online Library, and the MDPI database.
